# Measuring the optimistic bias of cross-validation in radiomics

**DOI:** 10.1038/s41598-026-62792-w

**Published:** 2026-07-20

**Authors:** Aydin Demircioğlu

**Affiliations:** Institute of Diagnostic and Interventional Radiology and Neuroradiology, University Hospital Essen, Hufelandstraße 55, 45147 Essen, Germany

**Keywords:** Radiomics, Cross-validation, Performance estimation, High-dimensional datasets, Bias, Computational biology and bioinformatics, Mathematics and computing, Medical research

## Abstract

In radiomics, independent external data are often unavailable and cross-validation (CV) schemes are widely used to obtain performance estimates. Simple k-fold CV is preferred over nested CV due to its lower computational cost, although nested CV is known to provide more accurate estimates. In a recent study, it was concluded that for practical reasons, there is no significant difference between the two schemes. However, that study was conducted only on low-dimensional datasets, which do not reflect typical radiomic data characteristics. While previous studies suggest that an optimistic bias, in which the true performance of the model is overestimated, might occur in high-dimensional datasets when only simple k-fold CV is used, the extent of this bias in radiomic datasets is currently unknown. This study evaluated whether nested CV is necessary in radiomics by comparing simple k-fold CV, holdout CV, and nested CV across 32 public radiomic datasets. In total, 30,720 models were evaluated to ensure a robust and comprehensive comparison. Each validation scheme was tested by repeatedly splitting the data using a 50:50 train-test ratio. The training data was used for model development and internal validation, whereas the test data was reserved for performance evaluation. Model performance was measured using the area under the receiver operating characteristic curve (AUC), F1-score, and Matthews correlation coefficient (MCC). Cross-validation estimates were then compared to the estimates on the test set to assess potential optimistic bias. In addition, the experiments were repeated on 110 low-dimensional datasets from the UCI repository, involving 105,600 models to compare findings between high- and low-dimensional data. The results revealed that in radiomic data, simple k-fold CV exhibited significant optimistic bias, with a bias of up to 0.128 in AUC, 0.130 in F1-score, and 0.264 in MCC, whereas holdout CV remained relatively unbiased. Nested CV showed low, but variable bias depending on the model, with a simple ensemble achieving the best performance. Larger sample sizes reduced both optimistic bias and variability, particularly in simple k-fold CV, but no significant relationship was observed with dataset dimensionality or feature count. In contrast, simple k-fold CV was unbiased on the UCI datasets (bias < 0.014 in AUC, < 0.016 in F1, and < 0.014 in MCC), and no clear differences were found between the three CV schemes. Therefore, in radiomics, there is a substantial practical difference between simple k-fold and nested CV. This finding further underscores that results from low-dimensional datasets do not necessarily generalize to radiomics.

**Supplementary Information:**

The online version contains supplementary material available at 10.1038/s41598-026-62792-w.

## Introduction

Radiomics is a quantitative, high-throughput approach for analyzing radiological images that systematically extracts large numbers of imaging features and applies machine learning to construct predictive models^1–3^. It is widely used in the development of clinical applications, spanning diagnosis, prognosis, and treatment planning^4–7^.

A major challenge in radiomics is the limited availability of large sample sizes, often due to factors such as annotation effort, rare diseases, and privacy constraints^8,9^. When combined with the typically high number of quantitative image features extracted from radiological data, these small sample sizes result in high-dimensional datasets in which the number of features often greatly exceeds the number of observations, a setting commonly referred to in machine learning as large-p, small-n (p » n). Such datasets pose challenges for machine learning modeling, as the high dimensionality increases the likelihood of identifying spurious patterns. This phenomenon, known as overfitting, can occur for several reasons. It may arise when small sample sizes cause a model to learn the inherent noise of the data rather than its predictive patterns^10,11^. It can also result from high model capacity, distribution shifts in the test data, or methodological issues such as data leakage^12^. The risk of overfitting is further exacerbated when model hyperparameters, such as the number of trees in a random forest, are tuned too aggressively, although hyperparameter optimization remains an essential step for achieving good performance^13^. Overfitted models typically perform well on training data but fail to generalize, which can result in a significant overestimation of actual performance on new data. This presents a major risk when such models are implemented in clinical practice. Therefore, to detect such an optimistic bias and obtain a reliable estimate of expected performance, an independent dataset is required for evaluation after training.

**Figure 1:**
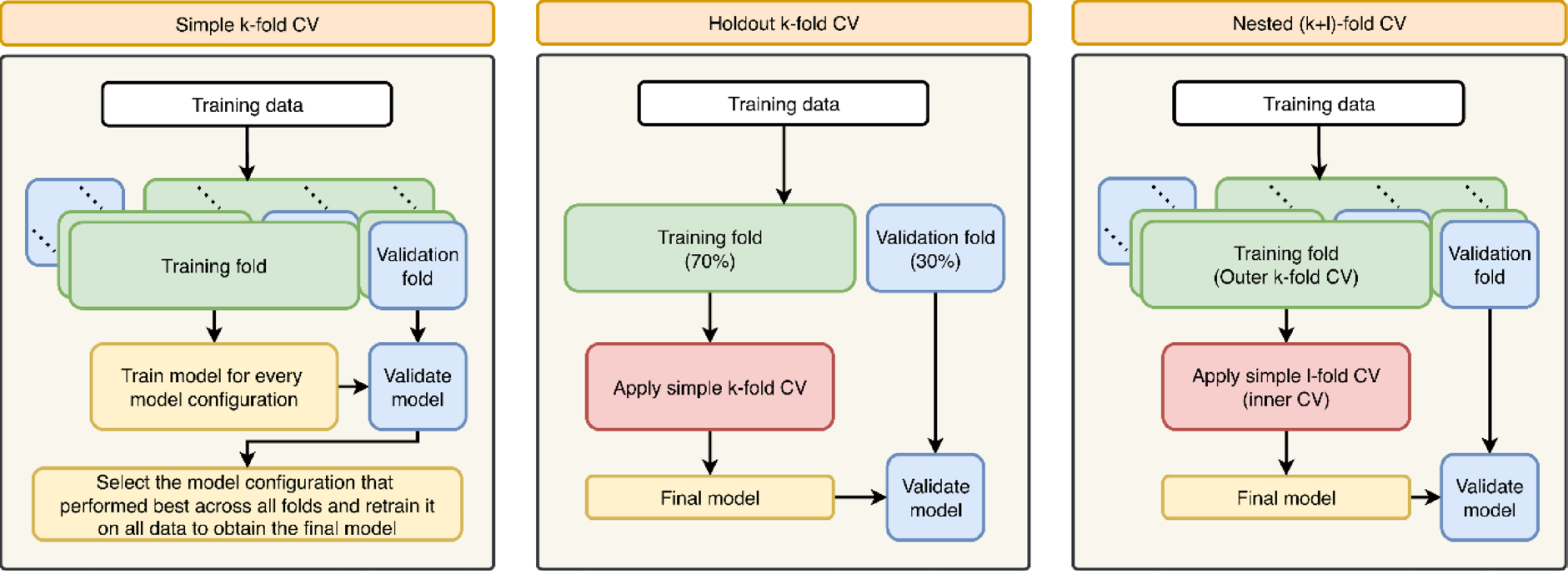
Often used validation schemes. In each validation scheme, the data is split into training and validation folds. Training is performed only on the training folds to avoid any data leakage. *Source*: Authors.

Since independent data, ideally from an external site, is often unavailable, validation schemes are commonly employed. A simple example is holdout validation, where a portion of the data is set aside before training and used exclusively to assess model performance after training, simulating the model’s behavior on new, unseen data^11^. However, this method can be considered as wasteful, as not all available data contribute to model training. Consequently, alternative validation strategies have been employed, with k-fold cross-validation (CV) being the most widely used^14,15^. Here, the dataset is systematically divided into k subsets (called folds), with each subset serving as the test set once while the remaining data are used for training (Fig. 1).

Despite its advantages, k-fold CV can introduce bias, i.e., overly optimistic performance estimates, when applied to models with numerous hyperparameters. This issue arises because hyperparameter tuning is performed on the same data used for validation, increasing the likelihood that a model is selected based on chance rather than its ability to generalize to new data^16,17^. To mitigate this, more advanced validation schemes have been developed. One such approach is holdout k-fold CV (Fig. 1), often referred to as k-fold CV with a holdout set. Similar to simple holdout validation, the dataset is split into a training and a holdout test set. Then, on the training set, a model is trained using simple k-fold CV, and the resulting model is then tested once on the holdout set to obtain an unbiased performance estimate.

Since holdout k-fold CV is as wasteful in data utilization as holdout validation, nested cross-validation^18^ has been proposed as an alternative (Fig. 1). This approach involves two CV loops: an outer CV that partitions the data for model evaluation and an inner CV used for hyperparameter tuning. Although nested CV provides a robust and unbiased assessment of model performance and is often considered the gold standard, it has a significantly higher computational cost and is therefore rarely used in radiomics. Additionally, unlike k-fold or holdout CV, nested CV is used solely for performance estimation and lacks a standardized approach for deriving a final model.

A recent study by Wainer and Cawley compared k-fold and nested CV, concluding that "for all practical purposes", there was no significant difference between the two methods^19^. However, their analysis was limited to datasets with low dimensionality from the UC Irvine Machine Learning Repository (UCI), where the number of samples exceeds the number of features. In contrast, radiomic datasets typically exhibit high dimensionality with highly correlated features, which can significantly affect model behavior^20^. While evidence from other studies suggests that performance overestimation is highly likely^17,21,22^. a more precise quantification of this optimistic bias has not yet been established in radiomics.

In this study, several validation methods were compared across a collection of 32 radiomic datasets. Performance estimates obtained during CV were evaluated using the area under the receiver operating characteristic curve (AUC), F1-score, and Matthews correlation coefficient (MCC), and compared with model performance on holdout test sets to assess potential bias. Additionally, the study investigated whether dataset characteristics such as the sample size or the number of features influenced observed differences. The analysis was also applied to low-dimensional datasets from the UCI repository to assess whether the observed patterns differed from the previously reported findings by Wainer and Cawley.

## Methods

This study exclusively utilized publicly available datasets that had received prior approval from their respective ethical review boards. Given its retrospective nature, the local Ethics Committee (Ethik-Kommission, Medizinische Fakultät der Universität Duisburg-Essen, Germany) waived the requirement for ethical approval.

### Datasets

This study employed the radMLBench collection, a repository of radiomic datasets^23^. Only datasets containing more than 100 samples were included (Table 1, Supplementary Materials, Table S1), because a minimum of 50 samples for model development is commonly recommended to ensure reliable estimates^24^. All datasets consisted of numeric features, and the target outcomes were binary. Prior to analysis, all features were standardized using z-scores.

**Table 1:** Radiomic datasets used in this study.

Dataset	Modality	Outcome	Number of samples	Number of Instances	Dimensionality	Balance	Source
Ahn2021	MRI	MGMT mutation status	114	108	0.96	55	https://doi.org/10.1016/j.ejrad.2019.108642
Arita2018	MRI	IDH mutation status	168	683	4.08	66	https://doi.org/10.1038/s41598-018-30273-4
BraTS-2021	MRI	MGMT mutation status	577	4060	7.04	52	https://doi.org/10.48550/arXiv.2107.02314
Dai2023	CT	Thrombocytopenia presence	119	851	7.17	26	https://doi.org/10.7717/peerj.16230
Deng2023	MRI	Pathological diagnosis	261	225	0.87	36	https://doi.org/10.1007/s13246-023-01300-0
Dong2022	CT	Tuberculosis granuloma presence	279	851	3.06	49	https://doi.org/10.7717/peerj.14127
Head-Neck-Radiomics-HN1	CT	Lymph node metastasis presence	137	105	0.78	45	http://doi.org/10.1038/ncomms5006
Hosny2018A	CT	Overall survival (at 2 years)	293	984	3.37	54	https://doi.org/10.1371/journal.pmed.1002711
Hosny2018B	CT	Overall survival (at 2 years)	207	984	4.76	29	https://doi.org/10.1371/journal.pmed.1002711
Hosny2018C	CT	Overall survival (at 2 years)	183	984	5.39	73	https://doi.org/10.1371/journal.pmed.1002711
Huang2023	CT	Malignancy presence	212	855	4.04	46	https://doi.org/10.1371/journal.pone.0292110
Hunter2023	CT	Nodule malignancy presence	520	1998	3.85	54	https://doi.org/10.1038/s41416-023-02480-y
ISPY1	MRI	Hormone receptor positive status	161	370	2.31	57	http://doi.org/10.7937/K9/TCIA.2016.HdHpgJLK
Keek2020	CT	Overall survival (at 3 years)	273	1322	4.85	44	https://doi.org/10.1371/journal.pone.0232639
LGG-1p19qDeletion	MRI	1p19q co-deletion status	159	2030	12.78	64	https://doi.org/10.1007/s10278-017-9984-3
LNDb	CT	Fleischner score group	173	105	0.62	66	https://doi.org/10.48550/arXiv.1911.08434
NSCLC-Radiogenomics	PET/CT	EGFR mutation status	144	105	0.74	16	http://doi.org/10.1148/radiol.12111607
PI-CAI	MRI	Gleason score risk group	969	3045	3.14	66	https://doi.org/10.1016/j.media.2021.102155
Petrillo2023	MRI	Luminal type presence	128	851	6.66	37	https://doi.org/10.1007/s11547-023-01718-2
Prostate-MRI-US-Biopsy	MRI	Gleason score risk group	773	1015	1.32	77	https://doi.org/10.1016/j.juro.2012.08.095
Sasaki2019	MRI	MGMT mutation status	138	587	4.27	49	https://doi.org/10.1038/s41598-019-50849-y
Song2020	MRI	Clinical significance	260	264	1.02	49	https://doi.org/10.1371/journal.pone.0237587
UCSF-PDGM	MRI	IDH mutation status	418	7105	17	89	https://doi.org/10.1148/ryai.220058
UPENN-GBM	MRI	MGMT mutation status	187	11,165	59.72	42	https://doi.org/10.1038/s41597-022-01560-7
Veeraraghavan2020	CT	High tumor burden presence	150	200	1.35	31	https://doi.org/10.1038/s41598-020-72475-9
WORC-Desmoid	MRI	Fibromatosis presence	203	1015	5.01	35	https://doi.org/10.48550/arXiv.2108.08618
WORC-GIST	CT	Gastrointestinal stromal tumors presence	245	1015	4.15	51	https://doi.org/10.48550/arXiv.2108.08618
WORC-Lipo	MRI	Malignancy presence	114	1015	8.92	50	https://doi.org/10.48550/arXiv.2108.08618
WORC-Liver	MRI	Malignancy presence	186	1015	5.47	51	https://doi.org/10.48550/arXiv.2108.08618
Zhang2023	CT	Histological invasiveness	203	1781	8.78	51	https://doi.org/10.7717/peerj.14559
Zhang2024A	PET/CT	Histological type	255	3850	15.11	57	https://doi.org/10.1371/journal.pone.0300170
Zhang2024B	CT	Lymph node metastasis presence	192	833	4.35	66	https://doi.org/10.7717/peerj.17111

Dimensionality denotes the ratio of number of features and samples. Balance denotes the percentage of positive outcomes compared to the total number of samples. BRAF: v-Raf Murine Sarcoma Viral Oncogene Homolog B1; CT: Computed Tomography; EGFR: Epidermal Growth Factor Receptor; IDH: Isocitrate Dehydrogenase; MGMT: O6-Methylguanine-DNA Methyltransferase; MRI: Magnetic Resonance Imaging; PET: Positron Emission Tomography.

### Study design

**Figure 2:**
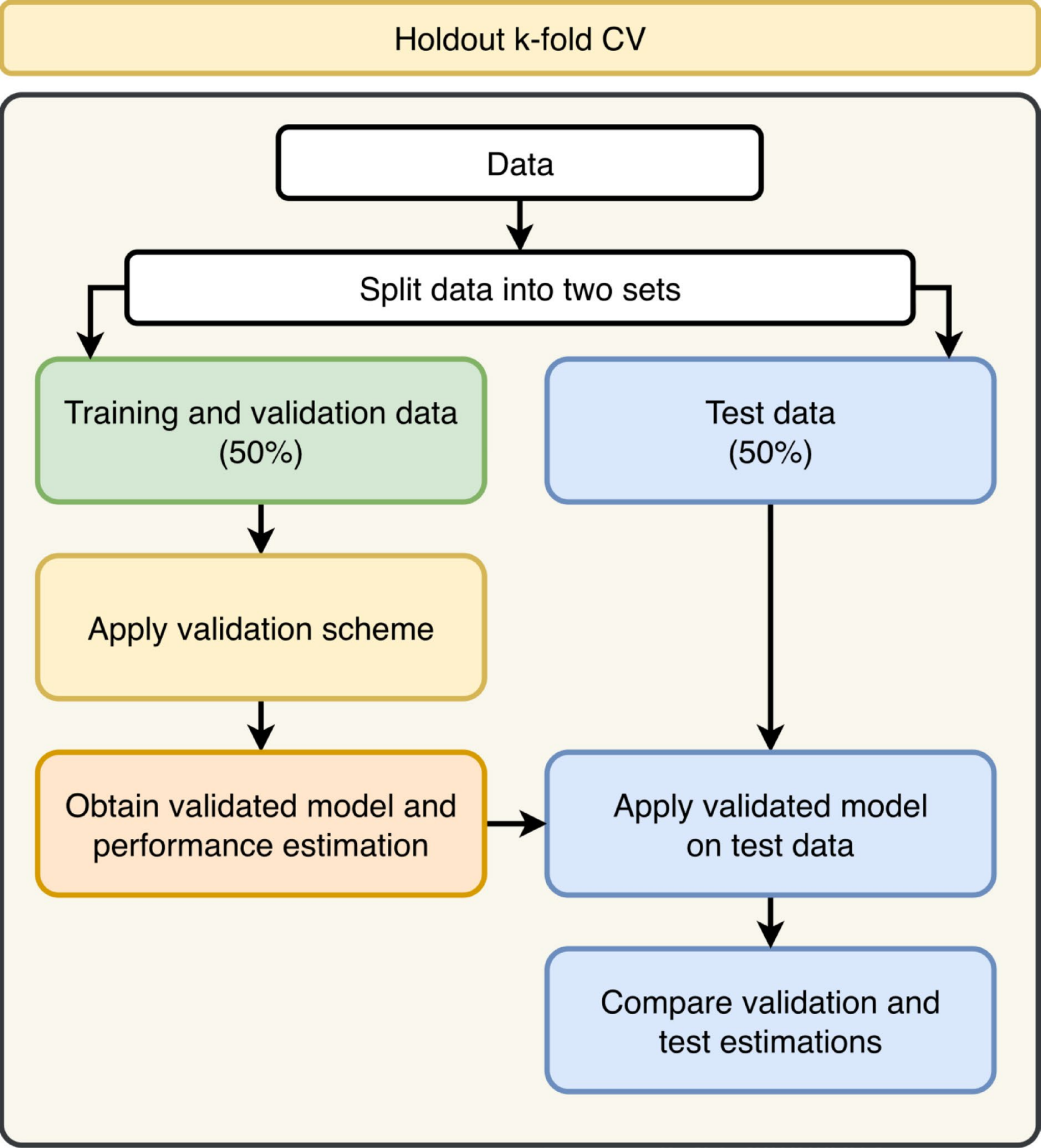
Overall study design. For each dataset, the data was split 50:50 using stratified splitting into a training set and a test set. A validation scheme was applied to the training data (also referred to as validation data), resulting in a performance estimate and a validated model. The model was subsequently applied once to the test set to obtain a final test estimate. The two estimates were then compared to identify whether any optimistic bias had taken place. To obtain a more robust comparison, the whole procedure was repeated 10 times for each dataset. *Source*: Authors.

The study followed the design outlined in Fig. 2. For each dataset, the data were first split into training and test sets using a 50:50 ratio, with stratification to maintain class distribution. This ratio was preferred over the more common 70:30 or 80:20 because, given the small sample sizes, those approaches would yield smaller test sets and consequently less stable evaluation metrics. On the training data, k-fold, holdout, or nested CV was applied to obtain performance estimates and a final model. This model was then tested once on the test set, and the resulting test performance was compared with the estimates obtained during validation. This entire procedure was repeated 10 times.

### Feature selection

Three algorithms commonly used in radiomics were employed^25,26^: Least Absolute Shrinkage and Selection Operator (LASSO), Minimum Redundancy Maximum Relevance ensemble (MRMRe), and Extramely Randomized Trees (ET). These algorithms do not perform a direct selection, but instead assign a score to each feature. Consequently, the number of top-scoring features to select was treated as a hyperparameter that needed to be tuned during CV. The number of selected features was chosen from N = 1, 2, 4, …, 32 (see Supplementary Materials, Table S2). Other hyperparameters such as the regularization parameter in LASSO were not considered and were left at their default values.

### Classifier

For classification, four algorithms were selected: Naive Bayes (NB), regularized logistic regression (LR), random forest (RF), and support vector machine (SVM). These algorithms have demonstrated strong performance in various benchmarks^19,27^. With the exception of NB, all classifiers have hyperparameters, some of which were selected for tuning during CV (see Supplementary Materials, Table S2). For LR and SVM, the regularization parameter C was tuned, while for the RF, the number of estimators and the maximum tree depth were optimized.

### Validation schemes

**Figure 3:**
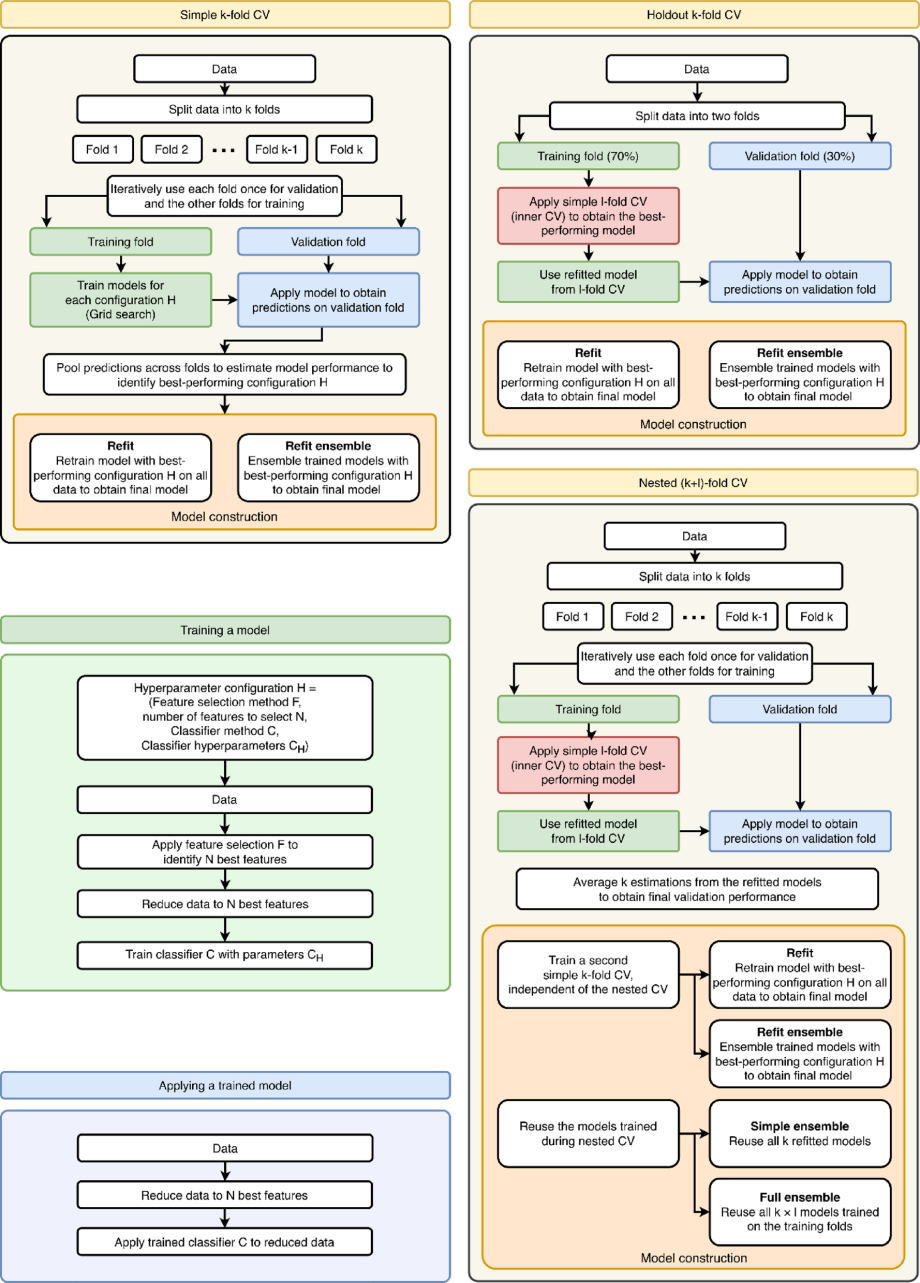
Schematical overview of all non-repeated validation schemes used. *Source*: Authors.

Three validation schemes were tested: simple k-fold, holdout k-fold, and nested CV (Fig. 3, Table 2). In simple k-fold CV, the entire dataset is used both to identify the best model and to estimate its performance. In holdout k-fold CV, the data are first split into a training and a held-out test set. Then, k-fold CV is applied to the training portion to identify the best model, which is subsequently evaluated on the test set. In nested CV, this procedure is repeated systematically in which an inner loop handles model tuning and an outer loop estimates performance. These two processes are kept strictly separated.

**Table 2:** Overview of all validation schemes used in this study.

Validation Scheme	Model construction	Number of trained models	Number of final models	Hyperparameter tuning	Final models have same configurations?	Performance estimation	Final model construction
Simple CV	Refit	k + 1	1	Grid search using k folds	Yes	Pooled predictions from k validation folds	Best configuration retrained on full training set
Simple CV	Ensemble	k	k	Grid search using k folds	Yes	Pooled predictions from k validation folds	All k models of the best configuration as a simple ensemble
Repeated Simple CV	Refit	k × r + 1	1	Grid search using k × r folds	Yes	Pooled predictions from k × r validation folds	Best configuration retrained once on full training set
Repeated Simple CV	Ensemble	k × r	k × r	Grid search using k × r folds	Yes	Pooled predictions from k × r validation folds	All k × r models of the best configuration as a simple ensemble
Holdout CV	Refit	k + 1	1	Grid search using k folds	Yes	Predictions on a single holdout fold	Best configuration retrained once on full training set
Holdout CV	Ensemble	k + 1	k	Grid search using k folds	Yes	Predictions on a single holdout fold	All k models of the best configuration as a simple ensemble
Repeated Holdout CV	Refit	k × r + 1	1	Grid search using k × r folds	Yes	Predictions on a single holdout fold	Best configuration retrained once on full training set
Repeated Holdout CV	Ensemble	k × r	k × r	Grid search using k × r folds	Yes	Predictions on a single holdout fold	All k × r models of the best configuration as a simple ensemble
Nested CV	Refit	k × (l + 1) + k + 1	1	Grid search using l folds, k times	Yes	Average of predictions on k validation folds	Best configuration retrained once on full training set
Nested CV	Refit ensemble	k × (l + 1) + k	k	Grid search using l folds, k times	Yes	Average of predictions on k validation folds	All k models of the best configuration (of the additional k-fold CV) as a simple ensemble
Nested CV	Simple ensemble	k × (l + 1)	k	Grid search using l folds, k times	No	Average of predictions on k validation folds	All k models retrained using the best configuration on the k-th fold as a simple ensemble
Nested CV	Full ensemble	k × (l + 1)	k × l	Grid search using l folds, k times	No	Average of predictions on k validation folds	All k × l models of the best configurations (on the k-th fold) as a simple ensemble
Repeated Nested CV	Refit	k × (l + 1) × r + k + 1	1	Grid search using l folds, k × r times	Yes	Average of predictions on k × r validation folds	Best configuration retrained once on full training set
Repeated Nested CV	Refit ensemble	k × (l + 1) × r + k	k	Grid search using l folds, k × r times	Yes	Average of predictions on k × r validation folds	All k models of the best configuration (of the additional k-fold CV) as a simple ensemble
Repeated Nested CV	Simple ensemble	k × (l + 1) × r	k × r	Grid search using l folds, k × r times	No	Average of predictions on k × r validation folds	All k × r models retrained using the best configuration on the folds as a simple ensemble
Repeated Nested CV	Full ensemble	k × (l + 1) × r	k × l × r	Grid search using l folds, k × r times	No	Average of predictions on k × r validation folds	All k × l × r models of the best configurations (on the k-th fold) as a simple ensemble

Number of final models: If the value is > 1 it means a simple ensemble is used for testing. If the column ‘Final models have same configurations?’ is ‘Yes’, then the models were obtained using the same feature selection method, the same number of best-performing features and the same classifier with the same hyperparameters. Therefore, these models differ only in the datasets on which they were trained.

Identification of the best model configuration was performed in all cases using a simple grid search, i.e., by evaluating all model configurations systematically on the training set. A model configuration was defined as a combination of a feature selection method F, the number of selected features N, a classifier C, and its corresponding hyperparameters C_H_. Given such a configuration H = (F, N, C, C_H_) and training data (Fig. 3), the model was constructed by first applying the feature selection F to the data to identify the N best-performing features. The data were then reduced by removing all other features. On this reduced dataset, the classifier C with hyperparameters C_H_ was trained (for example, this could be an SVM with C = 0.1). The trained model for the configuration H therefore comprised the set of top-N features and a trained classifier. Such a model was then applied to new data by first removing all features except the N features identified during training, and then applying the trained classifier C.

All validation schemes employed stratified splitting to maintain class distribution across folds, which is particularly important in presence of imbalanced class distributions. Stratification ensures that each fold reflects the overall prevalence of the positive class, preventing misleading performance estimates, particularly for metrics like the F1-score and MCC that are sensitive to class distribution. This also has clinical relevance, as a test set should reflect the class distribution likely encountered in clinical routine to ensure real-world applicability.

### Simple k-fold CV

Following the standard k-fold CV procedure, the data were divided into k folds using stratified splitting^11^. Sequentially, one fold was used for validation, while the remaining folds were utilized for training (Fig. 3). Hyperparameters were tuned using grid search, where for each model configuration H, a model was fitted on the training set and its predictions were then computed on the validation fold. These predictions were subsequently pooled across all validation folds. Then, to estimate the performance, the corresponding metric (AUC, MCC, or F1-score) was calculated. The configuration yielding the highest metric value was selected as the best-performing configuration.

A final model was then constructed by selecting the best-performing model configuration and retraining it on the entire training set. This model was referred to as the “k-fold CV, retrain.” Alternatively, instead of retraining, an ensemble model was constructed using all k models from the best-performing configuration from the CV procedure. Predictions from this ensemble model were obtained by averaging the probabilities. This model was referred to as the “k-fold CV, ensemble.”

### Holdout k-fold CV

In this scheme, a stratified holdout set was first set aside before applying simple k-fold CV to the remaining data (Fig. 3). After completing the CV process and obtaining the best model, its performance was estimated by evaluating it on the holdout data. Because the holdout data were never used for validation, they provide a less biased estimation than those obtained during CV. A final model is then constructed by refitting the best-performing configuration identified during the simple k-fold CV on the training folds on all data. This model is referred to as the “holdout k-fold CV, refit”. Similar to simple k-fold CV, an alternative model can be created via an ensemble of the k models obtained during CV, bypassing the refitting step. This model is therefore called the “holdout k-fold CV, ensemble” model. Note, however, that this ensemble uses only a portion of the data since the holdout data were not used to obtain the classifier.

### Nested CV

Nested CV is a two-tiered k-fold CV process applied to the data. In the outer loop, the data are partitioned into k stratified folds, with each fold sequentially serving as a test set (Fig. 3). On each outer training set, an inner l-fold CV is conducted, where the data are further divided into l stratified folds. Within the inner loop, l − 1 folds are used for training, while the remaining fold is used for model validation. This methodology is also referred to as (k × l)-fold CV.

Because a separate simple l-fold CV is conducted for each outer fold, the nested CV accordingly yields k different refitted models. These models are evaluated on the validation fold, and their average performance is taken as the final estimation. Since this approach may yield distinct best-performing models with different configurations, determining a single final model is not straightforward. While no universally accepted recommendation exists, often an additional, independent simple k-fold CV is applied to the data to identify a single optimal model configuration, which is then utilized to retrain a final model^19^. This approach is referred to herein as the “nested CV, refit” model. Instead of retraining a model with the best configuration determined in the additional k-fold CV, one can create an ensemble using the k refitted models from the independent k-fold CV, which is termed the “nested CV, refit ensemble” model.

Alternatively, one could forgo the additional k-fold cross-validation and directly ensemble the refitted models obtained from each of the k inner l-fold CV obtained during the nested CV procedure. This approach is referred to as the “nested CV, simple ensemble” model. Finally, an ensemble can be constructed using all k × l models trained during the nested CV, a method described as “nested CV, full ensemble” model.

### Repeated CV

The CV schemes can be applied in a repeated manner, which is expected to yield more accurate performance estimations and reduce bias. However, one must keep in mind that more models are trained, which in general will increase variance.

**Figure 4:**
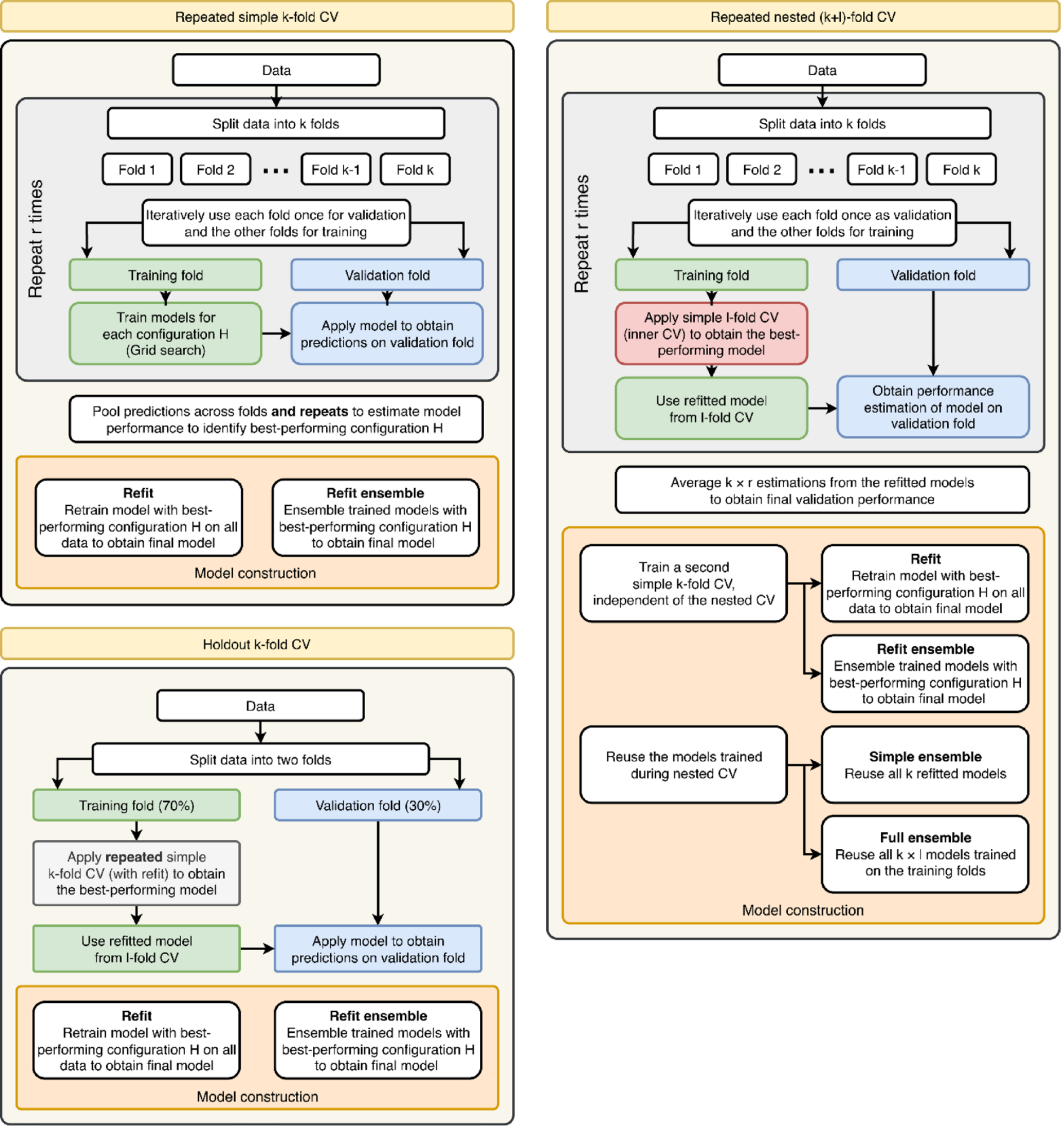
Schematical overview of all repeated validation schemes used. *Source*: Authors.

In repeated k-fold CV, the CV process is repeated r times, each with a different random split of the data (Fig. 4). The performance estimate for a given model configuration H is calculated by pooling across all repetitions (on the k × r validation folds). The final model configuration is selected as the one that achieves the highest performance. For the refit model, a single model is refitted on the entire data, while for the ensemble, the k × r trained models are used directly.

For repeated holdout k-fold CV, the repetitions are only applied to the simple k-fold CV, while the holdout set is kept the same (Fig. 4). In other words, a repeated k-fold simple CV is used on the training set, leading to a more accurate performance estimate. Accordingly, the final model is obtained in the same way as in case of the repeated simple k-fold CV, where it is constructed either by refitting a model with the obtained best-performing configuration H on the entire training data in case of the refit model, or by reusing the trained k × r models in the ensemble model.

Similarly, repeated nested CV can be performed by applying nested (k × l)-fold CV r times, each with different data splits (Fig. 4). The performance estimate is then obtained by averaging the estimates across all repetitions of the nested CV. The final model is then constructed similarly to the nested CV: one can apply an independent simple k-fold CV and use the resulting refitted model directly (refit) or via ensembling as final model (refit ensemble). Alternatively, one can utilize the trained models directly, either by ensembling the k × l refitted models obtained during the validation (simple ensemble) or by ensembling the full set of k × l × r trained best-performing models (full ensemble).

### Evaluation

The following validation schemes were evaluated: 5-fold CV, 10-fold CV, 5-fold holdout CV using a 30% holdout set, 10-fold holdout CV using a 30% holdout set, (5 × 10) nested CV, and (10 × 5) nested CV. Each scheme was also evaluated using five validation repetitions. The entire procedure was repeated 10 times for each dataset and each metric to obtain an estimate of the variation due to data splitting.

Being the de facto standard in radiomics, AUC was chosen as the primary performance metric. The AUCs obtained during CV, the validation estimates, were then compared against the estimations on the test set, called test estimates. The difference between these values was referred to as overestimation (or optimistic bias). Two additional metrics common in machine learning, F1-score and Matthews correlation coefficient (MCC), were employed. While F1 balances precision and recall and was briefly explored in the work of Wainer and Cawley^19^, MCC provides an alternative metric robust to class imbalance. Experiments using these metrics were performed independently, using a standard classification threshold of 0.5, and in each the corresponding metric was optimized during training.

Relative performance of each validation scheme was measured on each dataset by identifying the overall best-performing model across all repeats and schemes and using this value as a baseline for comparisons.

Additionally, the relationship between optimistic bias and dataset characteristics was examined. For each validation scheme and dataset, the average performance over all 10 repetitions was computed. These resulting metrics were then analyzed using linear regression against dataset characteristics, namely, the number of features, number of instances, and dimensionality. Bootstrapping was applied to estimate the uncertainty of this regression analysis.

To evaluate variation across repetitions, the standard deviation of the performance values across all 10 repetitions was computed for each dataset and validation scheme. These values were then averaged across all validation schemes and related to dataset characteristics using regression analysis.

Finally, computation times were assessed by measuring the total validation time, including data preparation but excluding the evaluation.

### Low-dimensional UCI datasets

In addition, a second set of datasets from the UCI repository, previously used by Wainer and Cawley^19^, was employed. To prevent computational issues, datasets with fewer than 100 samples or fewer than 20 positive instances were excluded. Datasets with more than 5000 samples were randomly subsampled. This resulted in a final collection of 110 low-dimensional datasets (see Supplementary Materials, Table S3). The same set of experiments was applied to this collection; however, since the datasets were low-dimensional, no feature selection step was performed (see Supplementary Materials, Table S4).

### Software

All modeling and training were performed using Python 3.10. Machine learning methods were implemented using scikit-learn v1.5.2. Grid search was conducted with Optuna v4.1.0. Further implementation details, all code, and results can be found in the GitHub repository at https://github.com/aydindemircioglu/radOverzealous.

### Statistical analysis

Descriptive statistics were reported as mean ± standard deviation. Statistical significance was defined as *p* < 0.05. All statistical analyses were conducted using the scipy module (v1.4.1). Bootstrapping was performed with 2000 repeats.

## Results

Overall, the evaluation on the radMLBench datasets encompassed 30,720 models, derived from 32 datasets, 32 validation schemes, 3 performance metrics, and 10 repeats (Tables 3, 4, 5). Similarly, 105,600 models were evaluated for the 110 UCI datasets.

**Table 3:** Overview of the performance estimates in AUC.

CV	Folds	Repeats	Model	AUC-Int	AUC-Test	Optimistic Bias	Relative Performance
Simple CV	5	1	Refit	0.754 ± 0.1	0.662 ± 0.144	0.092 ± 0.062	− 0.03 ± 0.011
			Ensemble	0.754 ± 0.1	0.637 ± 0.138	0.118 ± 0.054	− 0.057 ± 0.032
		5	Refit	0.731 ± 0.112	0.669 ± 0.148	0.062 ± 0.055	− 0.02 ± 0.01
			Ensemble	0.731 ± 0.112	0.638 ± 0.135	0.093 ± 0.042	− 0.052 ± 0.034
	10	1	Refit	0.763 ± 0.098	0.662 ± 0.143	0.102 ± 0.063	− 0.027 ± 0.014
			Ensemble	0.763 ± 0.098	0.635 ± 0.139	0.128 ± 0.057	− 0.058 ± 0.032
		5	Refit	0.744 ± 0.107	0.668 ± 0.148	0.076 ± 0.059	− 0.022 ± 0.012
			Ensemble	0.744 ± 0.107	0.634 ± 0.138	0.11 ± 0.045	− 0.055 ± 0.033
Holdout CV	5	1	Refit	0.658 ± 0.139	0.656 ± 0.138	0.002 ± 0.057	− 0.034 ± 0.018
			Ensemble	0.625 ± 0.125	0.625 ± 0.127	0.0 ± 0.041	− 0.064 ± 0.033
		5	Refit	0.666 ± 0.141	0.664 ± 0.146	0.001 ± 0.046	− 0.026 ± 0.016
			Ensemble	0.628 ± 0.127	0.619 ± 0.132	0.009 ± 0.048	− 0.068 ± 0.033
	10	1	Refit	0.648 ± 0.138	0.653 ± 0.141	− 0.005 ± 0.042	− 0.037 ± 0.02
			Ensemble	0.635 ± 0.125	0.626 ± 0.133	0.009 ± 0.041	− 0.065 ± 0.032
		5	Refit	0.652 ± 0.138	0.66 ± 0.145	− 0.008 ± 0.053	− 0.03 ± 0.016
			Ensemble	0.635 ± 0.134	0.63 ± 0.14	0.005 ± 0.043	− 0.06 ± 0.032
Nested CV	5 × 10	1	Refit	0.662 ± 0.135	0.662 ± 0.144	− 0.0 ± 0.032	− 0.038 ± 0.018
			Refit ensemble	0.662 ± 0.135	0.637 ± 0.138	0.025 ± 0.025	− 0.062 ± 0.032
			Simple ensemble	0.662 ± 0.135	0.673 ± 0.149	− 0.011 ± 0.034	− 0.015 ± 0.011
			Full ensemble	0.637 ± 0.124	0.658 ± 0.146	− 0.022 ± 0.038	− 0.031 ± 0.019
		5	Refit	0.662 ± 0.136	0.669 ± 0.148	− 0.007 ± 0.032	− 0.028 ± 0.014
			Refit ensemble	0.662 ± 0.136	0.638 ± 0.135	0.024 ± 0.037	− 0.065 ± 0.038
			Simple ensemble	0.662 ± 0.136	0.681 ± 0.152	− 0.019 ± 0.035	− 0.009 ± 0.015
			Full ensemble	0.635 ± 0.129	0.671 ± 0.151	− 0.036 ± 0.044	− 0.019 ± 0.016
	10 × 5	1	Refit	0.667 ± 0.133	0.662 ± 0.143	0.005 ± 0.031	− 0.033 ± 0.016
			Refit ensemble	0.667 ± 0.133	0.635 ± 0.14	0.032 ± 0.031	− 0.057 ± 0.034
			Simple ensemble	0.667 ± 0.133	0.68 ± 0.148	− 0.013 ± 0.04	− 0.011 ± 0.012
			Full ensemble	0.639 ± 0.135	0.666 ± 0.15	− 0.027 ± 0.041	− 0.025 ± 0.017
		5	Refit	0.666 ± 0.132	0.668 ± 0.148	− 0.001 ± 0.032	− 0.028 ± 0.015
			Refit ensemble	0.666 ± 0.132	0.634 ± 0.138	0.032 ± 0.035	− 0.059 ± 0.037
			Simple ensemble	0.666 ± 0.132	0.681 ± 0.152	− 0.015 ± 0.042	− 0.009 ± 0.016
			Full ensemble	0.639 ± 0.13	0.673 ± 0.152	− 0.034 ± 0.048	− 0.018 ± 0.018

For k-fold CV, performance estimation is independent of model creation. While refit and refit ensembles for nested CV both use another internal k-fold CV for the model creation, this model uses a different split, thus the estimates are not the same as those of the k-fold CV.

**Table 4:** Overview of the performance estimates in F1-score.

CV	Folds	Repeats	Model	F1-Int	F1-Test	Optimistic Bias	Relative Performance
Simple CV	5	1	Refit	0.734 ± 0.141	0.649 ± 0.182	0.084 ± 0.048	− 0.033 ± 0.024
			Ensemble	0.734 ± 0.141	0.617 ± 0.219	0.117 ± 0.09	− 0.066 ± 0.061
		5	Refit	0.71 ± 0.154	0.666 ± 0.169	0.044 ± 0.029	− 0.017 ± 0.018
			Ensemble	0.71 ± 0.154	0.613 ± 0.233	0.097 ± 0.098	− 0.07 ± 0.085
	10	1	Refit	0.741 ± 0.137	0.646 ± 0.188	0.095 ± 0.06	− 0.037 ± 0.031
			Ensemble	0.741 ± 0.137	0.611 ± 0.225	0.13 ± 0.102	− 0.071 ± 0.074
		5	Refit	0.72 ± 0.147	0.659 ± 0.176	0.061 ± 0.038	− 0.024 ± 0.021
			Ensemble	0.72 ± 0.147	0.611 ± 0.229	0.109 ± 0.097	− 0.072 ± 0.077
Holdout CV	5	1	Refit	0.629 ± 0.204	0.631 ± 0.2	− 0.001 ± 0.038	− 0.052 ± 0.041
			Ensemble	0.594 ± 0.231	0.601 ± 0.225	− 0.007 ± 0.045	− 0.081 ± 0.072
		5	Refit	0.647 ± 0.188	0.647 ± 0.187	− 0.001 ± 0.046	− 0.035 ± 0.03
			Ensemble	0.593 ± 0.228	0.602 ± 0.225	− 0.009 ± 0.024	− 0.081 ± 0.077
	10	1	Refit	0.632 ± 0.193	0.639 ± 0.198	− 0.007 ± 0.037	− 0.043 ± 0.038
			Ensemble	0.608 ± 0.222	0.603 ± 0.22	0.005 ± 0.032	− 0.079 ± 0.061
		5	Refit	0.635 ± 0.198	0.64 ± 0.198	− 0.004 ± 0.045	− 0.043 ± 0.038
			Ensemble	0.592 ± 0.232	0.599 ± 0.226	− 0.007 ± 0.037	− 0.084 ± 0.073
Nested CV	5 × 10	1	Refit	0.637 ± 0.184	0.651 ± 0.181	− 0.014 ± 0.038	− 0.032 ± 0.025
			Refit ensemble	0.637 ± 0.184	0.617 ± 0.217	0.02 ± 0.061	− 0.065 ± 0.06
			Simple ensemble	0.637 ± 0.184	0.652 ± 0.199	− 0.015 ± 0.04	− 0.031 ± 0.037
			Full ensemble	0.597 ± 0.225	0.598 ± 0.264	− 0.001 ± 0.057	− 0.085 ± 0.105
		5	Refit	0.638 ± 0.187	0.666 ± 0.169	− 0.028 ± 0.029	− 0.017 ± 0.018
			Refit ensemble	0.638 ± 0.187	0.613 ± 0.233	0.025 ± 0.082	− 0.07 ± 0.085
			Simple ensemble	0.638 ± 0.187	0.66 ± 0.192	− 0.023 ± 0.027	− 0.022 ± 0.029
			Full ensemble	0.599 ± 0.226	0.605 ± 0.276	− 0.005 ± 0.07	− 0.078 ± 0.118
	10 × 5	1	Refit	0.639 ± 0.192	0.645 ± 0.188	− 0.007 ± 0.03	− 0.037 ± 0.031
			Refit ensemble	0.639 ± 0.192	0.609 ± 0.225	0.029 ± 0.065	− 0.073 ± 0.074
			Simple ensemble	0.639 ± 0.192	0.66 ± 0.19	− 0.021 ± 0.03	− 0.023 ± 0.024
			Full ensemble	0.595 ± 0.233	0.599 ± 0.27	− 0.004 ± 0.06	− 0.084 ± 0.111
		5	Refit	0.636 ± 0.195	0.659 ± 0.176	− 0.023 ± 0.039	− 0.024 ± 0.021
			Refit ensemble	0.636 ± 0.195	0.612 ± 0.229	0.025 ± 0.073	− 0.071 ± 0.077
			Simple ensemble	0.636 ± 0.195	0.663 ± 0.188	− 0.027 ± 0.031	− 0.019 ± 0.023
			Full ensemble	0.598 ± 0.231	0.607 ± 0.275	− 0.009 ± 0.07	− 0.075 ± 0.117

For k-fold CV, performance estimation is independent of model creation. While refit and refit ensembles for nested CV both use another internal k-fold CV for the model creation, this model uses a different split, thus the estimates are not the same as those of the k-fold CV.

**Table 5:** Overview of the performance estimates in MCC.

CV	Folds	Repeats	Model	MCC-Int	MCC-Test	Optimistic Bias	Relative Performance
Simple CV	5	1	Refit	0.443 ± 0.184	0.241 ± 0.24	0.201 ± 0.09	− 0.059 ± 0.034
			Ensemble	0.443 ± 0.184	0.191 ± 0.227	0.252 ± 0.091	− 0.11 ± 0.082
		5	Refit	0.38 ± 0.204	0.254 ± 0.243	0.127 ± 0.069	− 0.047 ± 0.029
			Ensemble	0.38 ± 0.204	0.191 ± 0.221	0.189 ± 0.071	− 0.109 ± 0.084
	10	1	Refit	0.456 ± 0.182	0.25 ± 0.239	0.206 ± 0.094	− 0.05 ± 0.026
			Ensemble	0.456 ± 0.182	0.192 ± 0.233	0.264 ± 0.094	− 0.108 ± 0.075
		5	Refit	0.404 ± 0.198	0.257 ± 0.244	0.146 ± 0.082	− 0.043 ± 0.028
			Ensemble	0.404 ± 0.198	0.202 ± 0.237	0.202 ± 0.077	− 0.099 ± 0.067
Holdout CV	5	1	Refit	0.237 ± 0.256	0.239 ± 0.247	− 0.002 ± 0.06	− 0.061 ± 0.037
			Ensemble	0.173 ± 0.217	0.169 ± 0.223	0.004 ± 0.077	− 0.131 ± 0.083
		5	Refit	0.252 ± 0.232	0.245 ± 0.247	0.007 ± 0.065	− 0.055 ± 0.037
			Ensemble	0.17 ± 0.204	0.171 ± 0.217	− 0.001 ± 0.058	− 0.129 ± 0.076
	10	1	Refit	0.245 ± 0.231	0.244 ± 0.24	0.001 ± 0.076	− 0.057 ± 0.041
			Ensemble	0.192 ± 0.211	0.173 ± 0.223	0.019 ± 0.075	− 0.127 ± 0.072
		5	Refit	0.248 ± 0.222	0.244 ± 0.245	0.003 ± 0.065	− 0.056 ± 0.032
			Ensemble	0.194 ± 0.207	0.179 ± 0.23	0.014 ± 0.07	− 0.121 ± 0.068
Nested CV	5 × 10	1	Refit	0.249 ± 0.232	0.242 ± 0.242	0.007 ± 0.048	− 0.058 ± 0.033
			Refit ensemble	0.249 ± 0.232	0.19 ± 0.226	0.059 ± 0.083	− 0.11 ± 0.083
			Simple ensemble	0.249 ± 0.232	0.271 ± 0.253	− 0.022 ± 0.054	− 0.029 ± 0.03
			Full ensemble	0.183 ± 0.213	0.217 ± 0.245	− 0.034 ± 0.065	− 0.083 ± 0.08
		5	Refit	0.247 ± 0.235	0.254 ± 0.243	− 0.007 ± 0.035	− 0.047 ± 0.029
			Refit ensemble	0.247 ± 0.235	0.192 ± 0.222	0.055 ± 0.077	− 0.108 ± 0.084
			Simple ensemble	0.247 ± 0.235	0.278 ± 0.251	− 0.031 ± 0.045	− 0.022 ± 0.029
			Full ensemble	0.186 ± 0.215	0.226 ± 0.247	− 0.04 ± 0.065	− 0.075 ± 0.08
	10 × 5	1	Refit	0.242 ± 0.237	0.25 ± 0.24	− 0.008 ± 0.038	− 0.05 ± 0.026
			Refit ensemble	0.242 ± 0.237	0.193 ± 0.232	0.049 ± 0.069	− 0.108 ± 0.071
			Simple ensemble	0.242 ± 0.237	0.271 ± 0.253	− 0.03 ± 0.052	− 0.029 ± 0.033
			Full ensemble	0.183 ± 0.214	0.226 ± 0.245	− 0.042 ± 0.066	− 0.074 ± 0.084
		5	Refit	0.254 ± 0.233	0.258 ± 0.243	− 0.004 ± 0.048	− 0.043 ± 0.028
			Refit ensemble	0.254 ± 0.233	0.201 ± 0.236	0.052 ± 0.07	− 0.099 ± 0.067
			Simple ensemble	0.254 ± 0.233	0.276 ± 0.25	− 0.023 ± 0.052	− 0.024 ± 0.03
			Full ensemble	0.192 ± 0.216	0.234 ± 0.248	− 0.043 ± 0.072	− 0.066 ± 0.08

For k-fold CV, performance estimation is independent of model creation. While refit and refit ensembles for nested CV both use another internal k-fold CV for the model creation, this model uses a different split, thus the estimates are not the same as those of the k-fold CV.

**Figure 5:**
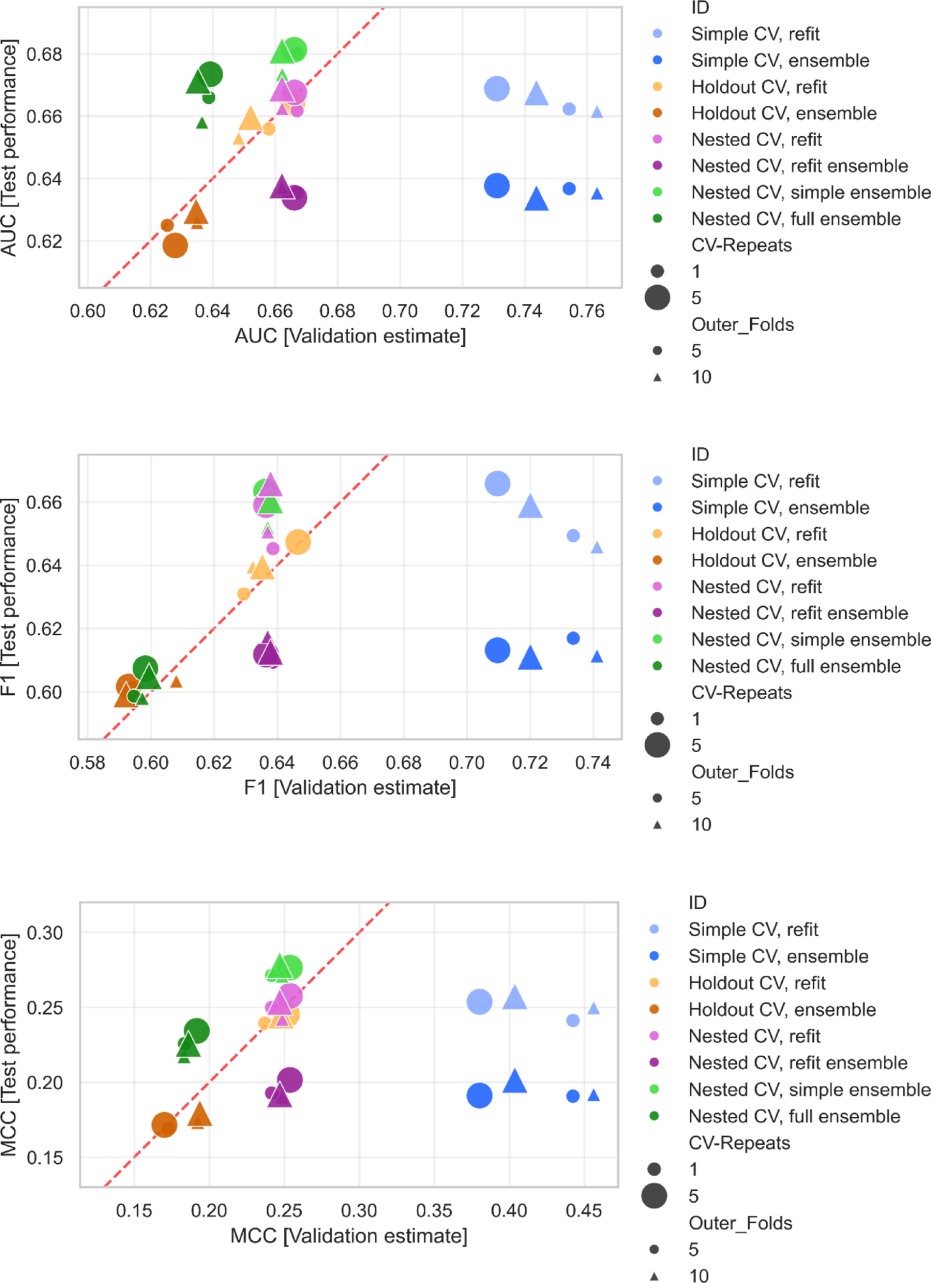
Scatter plots comparing validation and test estimates (amount of overestimation) for AUC, F1-scores, and MCC. CV schemes below the dashed line indicate overestimation, while those above exhibit better performance than expected. Schemes positioned higher in the plot demonstrate better performance on the test set, whereas those positioned lower exhibit poorer performance. *Source*: Authors.

The results showed that simple k-fold CV is strongly biased and can exhibit an optimistic bias of up to 0.128 ± 0.057 in AUC, up to 0.13 ± 0.102 in F1, and up to 0.264 ± 0.094 in MCC, depending on the validation scheme and dataset (Fig. 5). In contrast, across all metrics, holdout CV is relatively unbiased. Nested CV may show some minor bias, either positive or negative, depending on the model used for evaluation.

Across all metrics, the relative performance of the refit models was better than the ensemble models for simple and holdout CV, suggesting that ensembling models with the same model configuration does not improve performance (Fig. 5). In contrast, for nested CV, the refit ensemble performed the worst, while the refit models and full ensemble performed better. The best results were achieved with the simple ensemble.

Repeating the CV five times to identify the best model configuration helped reduce the overestimation in simple k-fold CV (reduction between 0.018 and 0.030 in AUC, 0.020–0.040 in F1, and 0.060–0.074 in MCC), though it remained large in absolute terms. For other CV schemes, the reduction was smaller (up to 0.014 in AUC, up to 0.016 in F1, and 0.014 in MCC). Increasing the number of outer folds from five to ten had a stronger effect on simple k-fold CV (between 0.014 and 0.017 in AUC, 0.012–0.017 in F1, and 0.013–0.019 in MCC), and a smaller effect in the other CV schemes (0.002–0.009 in AUC, up to 0.012 in F1, and 0.003–0.015 in MCC).

**Figure 6:**
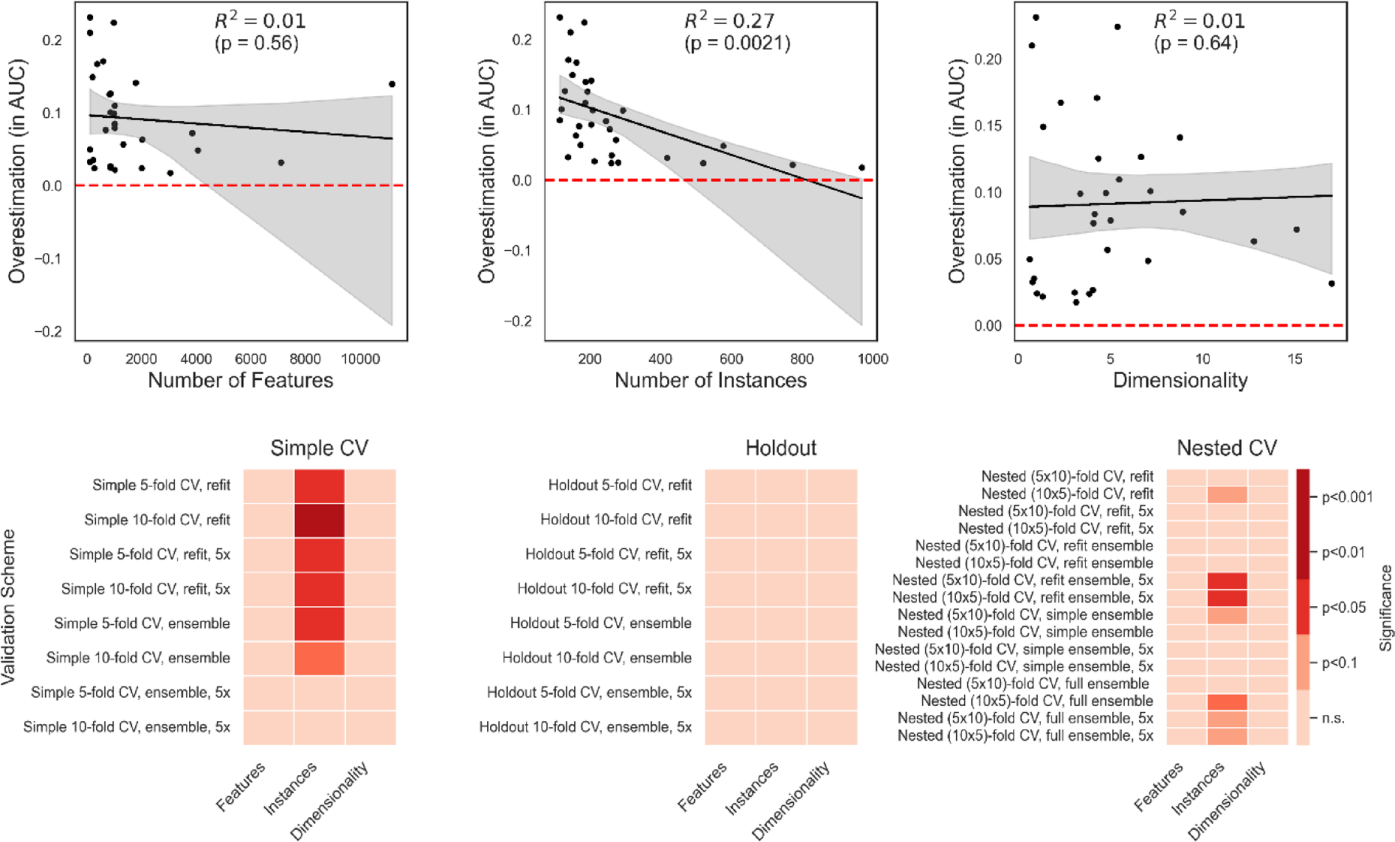
Association between the amount of overestimation in AUC and dataset characteristics. Top row: Scatter plots for simple 5-fold CV demonstrating a significant association between the amount of overestimation in AUC and the number of instances. Bottom row: Heatmaps summarizing the statistical significance (p-values) of the association between overestimation and dataset characteristics across all validation schemes. The three scatter plots shown in the top row correspond to the first row (‘Simple 5-fold CV, refit’) of the ‘Simple CV’ heatmap. For improved clarity, two datasets, Brancato2023 and UPENN-GBM, both of which have a dimensionality greater than 40, were excluded from the plots involving dimensionality. *Source*: Authors.

No significant relationship between optimistic bias and the number of features or dimensionality was found across all metrics. However, the number of instances significantly influenced it in simple k-fold CV in nearly all schemes examined, regardless of the metric (Fig. 6), whereas for nested CV, significant effects were observed only in a few cases (see Supplementary Materials, Fig. S1, S2).

**Figure 7:**
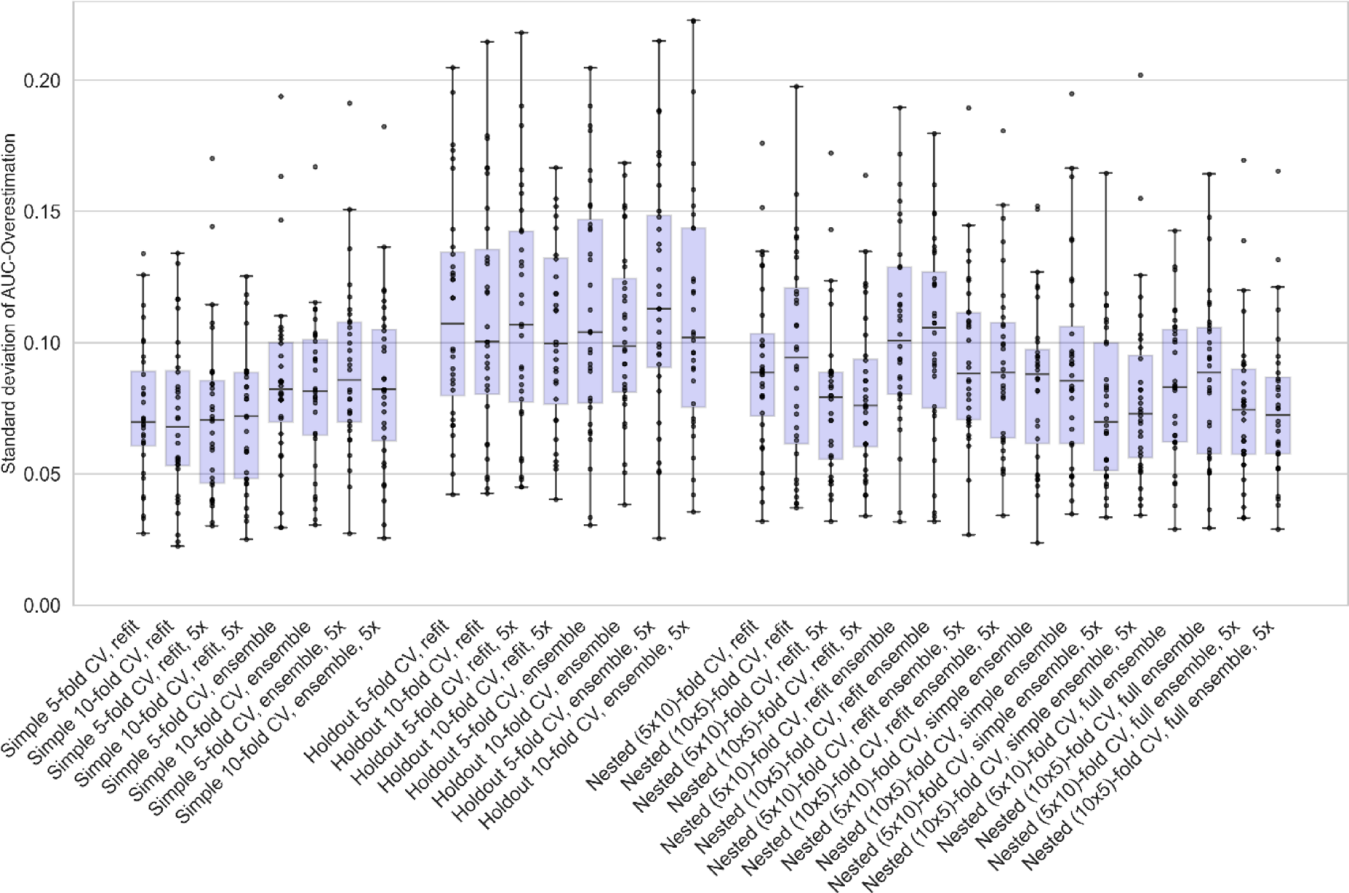
Graphical boxplot of the standard deviation of the amount of overestimation in AUC. For each dataset and validation scheme, the standard deviation across the 10 repeats was averaged and plotted. *Source*: Authors.

**Figure 8:**
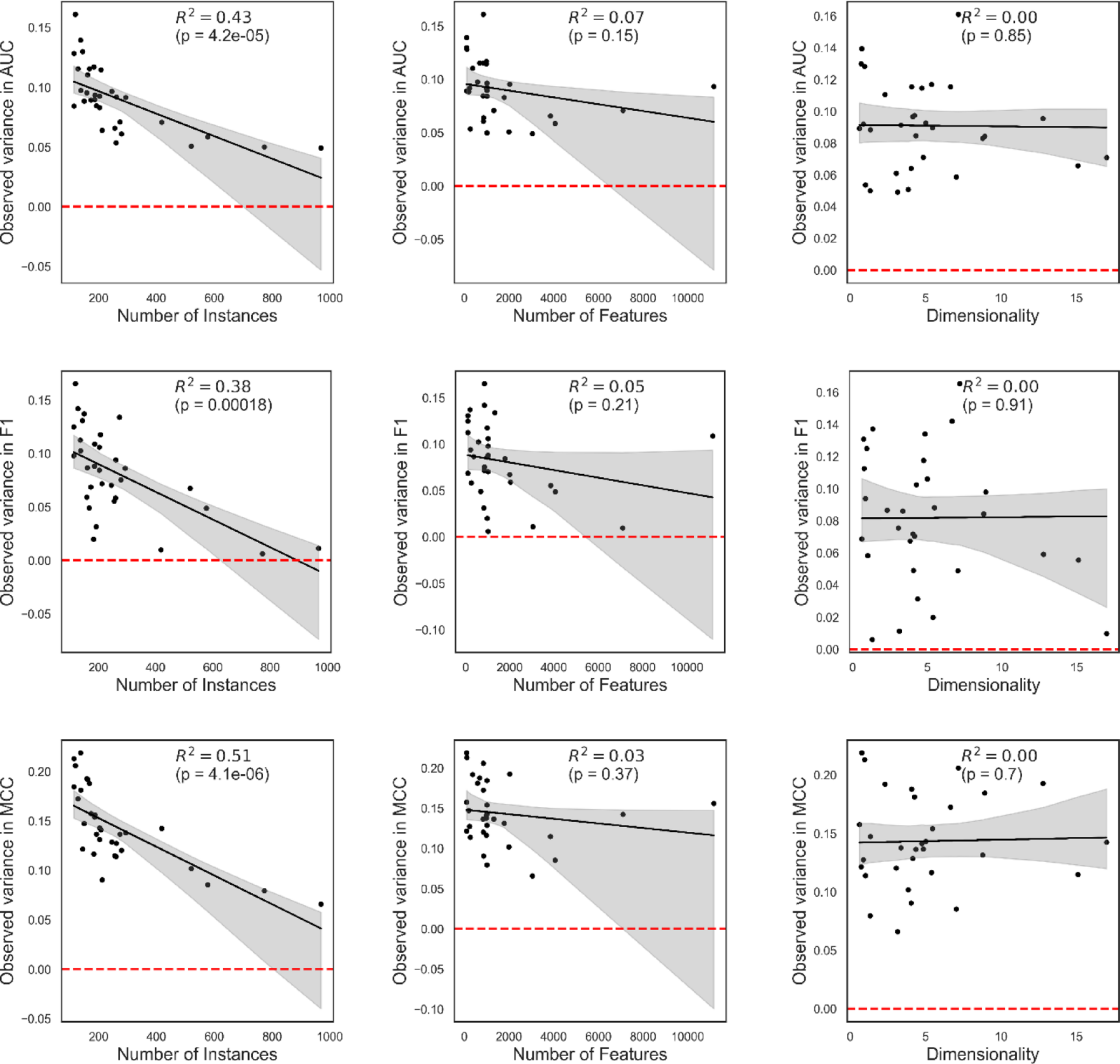
Association between the standard deviation of the amount of overestimation in AUC and dataset characteristics. A significant association was observed between the amount of overestimation across all metrics and the number of instances, but not with the number of features or dimensionality. For improved clarity, two datasets, Brancato2023 and UPENN-GBM, both of which have a dimensionality greater than 40, were excluded from the plots involving dimensionality. *Source*: Authors.

Observed variance was generally high across all validation schemes, with holdout CV showing notably more variance (Fig. 7, Supplementary Materials, Fig. S3, S4). Variance was associated with sample size, being higher for smaller datasets, but did not depend on the number of features or dimensionality (Fig. 8).

As expected, computation times increased with the number of models trained during validation (Supplementary Materials, Fig. S5). For example, simple 5-fold CV involved the training of five models while, a (5 × 10) nested CV trained 50 models, resulting in ten times longer computational time.

The same experiments on the UCI data collection revealed no clear difference between the three CV methods. On average, no overestimation was observed for simple k-fold CV compared to radiomic datasets in AUC (0.008 vs 0.128), and only minor bias in F1 (0.021 vs 0.13) and in MCC (0.038 vs 0.264) (see Supplementary Materials, Table S5-S7, Fig. S6-S8). A similar pattern emerged with respect to refit and ensemble models: refit models were less biased than ensemble models, with the simple ensemble performing best. Repeating the CV or increasing the number of outer folds had little effect on the results of the three CV schemes (< 0.004 difference in AUC, < 0.008 in F1, and < 0.014 in MCC). While some significant relationships between the amount of overestimation and the number of instances and dimensionality were found, especially for simple k-fold CV, no such relationships were observed for the number of features (Supplementary Materials, Fig. S9-S11). Across all metrics, the observed variance was generally much lower across all validation schemes and appeared to decrease with an increasing number of instances and features, while increasing with dimensionality (Supplementary Materials, Fig. S12-S17). Computational times were substantially lower (roughly 8 times less), highlighting a clear difference between low- and high-dimensional dataset collections (Supplementary Materials, Fig. S18).

## Discussion

In radiomics, cross-validation is commonly used to estimate performance due to the lack of independent test data. This study compared several validation schemes across a large set of radiomic datasets and showed that in contrast to other schemes, simple k-fold CV is largely biased and cannot replace nested CV in radiomics.

The bias of the simple k-fold CV is very likely due to the use of the same data for model selection and performance estimation. This observed overestimation is one reason why it is rarely used in radiomic studies^14,15^. In contrast, holdout CV yielded rather unbiased estimates as it uses an independent test set for estimation. Surprisingly, nested CV yielded unbiased estimates only when a second simple k-fold CV with refit was used to obtain a final model, particularly for AUC and F1 scores.

Ensemble models for simple and holdout CV underperformed, likely due to the reliance of ensembling on independent model errors^28,29^. The models for these two validation schemes were trained with identical hyperparameters, which potentially introduced dependencies among their errors. This outcome was not entirely predictable a priori, as small sample sizes and consequent high model variance could have mitigated this effect.

With nested CV, the situation differed. By design, the models trained within each inner loop can vary substantially in their configuration. Consequently, the simple ensemble exhibited improved overall performance. The other approaches, however, did not fare as well. As the refit ensemble was trained using a standard k-fold CV procedure, its underperformance mirrored that of the simple CV ensemble. In contrast, the full ensemble comprises both partially dependent and independent models. This resulted in lower validation estimates; however, performance on unseen data improved, surpassing that of the refit ensemble for AUC and MCC, while F1 remained comparable.

Given the dependence of the estimates on the data split, quantified by the standard deviation, substantial variations were observed. These variations, however, did not exhibit a strong dependence on the validation scheme, although holdout CV showed marginally larger variations across all metrics, potentially attributable to the reduced training set size, comprising 70% of the training data.

Analysis of the association between data characteristics and the amount of overestimation revealed a pronounced effect of sample size in k-fold CV and, to a lesser extent, in nested CV. The results suggested that larger sample sizes could mitigate the optimistic bias in k-fold CV. However, due to the limited number of such datasets included in this study, this conclusion remains tentative.

These results are consistent with those reported by Wainer and Cawley^19^. To eliminate any potential bias from differing experimental setups, the experiments were rerun using their same dataset collection from UCI. The results confirm that, for low-dimensional datasets, simple CV and nested CV do not differ significantly in terms of predictive performance, particularly regarding AUC. While a slightly larger discrepancy was observed for F1 and MCC than reported by Wainer and Cawley, it is important to note that their analysis of these metrics was limited to only the best-performing classifiers^19^. These findings clearly demonstrate that results obtained from low-dimensional datasets are not always applicable to radiomic datasets. They also further establish that in radiomics, simple k-fold CV is inadequate due to the substantial bias, and nested CV should be employed instead.

One of the first studies to highlight the optimization bias of simple k-fold CV in high-dimensional datasets was conducted by Varma and Simon^17^. They demonstrated that using simple k-fold CV for both parameter tuning and error estimation yields a severe overestimation of performance metrics, and they established that nested CV can yield an almost unbiased estimation of true model performance.

However, Subramanian and Simon pointed out that it is not only the high dimensionality of the datasets that leads to a substantial overestimation^21^. Using simulated datasets, they observed optimistic bias even in low-dimensional datasets, especially when the relationship of the outcome to the predictor variables is weak. Indeed, the results presented in this study underline this problem in radiomics. The experiments show a mean AUC of around 0.66, which indicates a weaker relationship and thus a stronger overestimation problem than that observed on the low-dimensional datasets of the UCI collection, where a mean AUC of 0.89 was found.

Vabalas et al. further underscored the issue of overestimation bias by demonstrating that simple k-fold CV introduces a more substantial overestimation of performance when applied to smaller datasets^22^. By using synthetic datasets, they highlighted that while larger sample sizes reduce this optimistic bias, a substantial amount remains evident even for datasets with more than a thousand samples. This is in line with the results presented in this study. Given that typical radiomic datasets contain only a few hundred samples, their findings underline the importance of quantitatively measuring this overestimation bias within radiomics workflows, as investigated in this study.

In a similar study, Singh et al. investigated various validation strategies and their variability across different random seeds, observing that a simple train-test split yielded substantial variation and could generate spurious false positives, whereas 10-fold CV with 10 repeats or bootstrapping exhibited considerably less variation^30^. However, their analysis was limited to two low-dimensional datasets, thus limiting the generalizability of their findings to radiomic data. In a separate benchmarking study, Borra and Di Ciaccio compared several validation methods on several synthetic datasets and also arrived at the conclusion that for small sample sizes validation methods tend to overfit^31^.

These previous findings clearly demonstrate the optimistic bias of simple k-fold CV in general machine learning contexts, especially for low-dimensional and synthetic datasets. The presented study builds upon these findings by quantifying these effects across a large collection of real-world radiomic datasets. Indeed, the combination of high dimensionality, highly correlated features, and small sample sizes typical of radiomic datasets makes the overestimation there more severe, making a quantification of this bias highly necessary. By comparing different validation schemes and ensembling methods on real radiomic data, this work also highlights that the choice of a validation scheme is a key factor in radiomics. It also offers an explanation for why some radiomics models perform well in studies but fail to replicate on unseen data, which currently obstructs the clinical application of radiomics. The optimistic bias in radiomic studies is further deepened if feature selection is not fully nested within the CV. Krawczuk and Łukaszuk considered the bias arising from applying feature selection to all data in high-dimensional genetic datasets, and they observed a large optimistic bias^32^. Subsequently, the same problem was also found in radiomics, where a similar bias was reported^12^.

The aim of applying CV in radiomics is to estimate how well a model generalizes to unseen data, as would be encountered in clinical routine. However, other challenges that obstruct this goal persist and affect nearly every part of radiomics, limiting the reproducibility of its findings^33,34^. For example, image acquisition generally depends strongly on imaging parameters, which are often vendor-specific and thus not compatible across scanners. The extraction of radiomics features also depends on parameters, which are often chosen ad hoc. Extracted features depend on segmentations, and slight differences might lead to non-reproducible results in the worst case. Recommendations for many of these steps have been proposed by the International Biomarker Standardization Initiative (IBSI), but these are not yet complete, and not all software packages follow the current standards in full^35^. Furthermore, to obtain unbiased estimates, in all steps of model development one must ensure that no data leakage occurs, and that the model is robust, reproducible, and compared against a reasonable baseline^36,37^.

Accordingly, while this study demonstrates that simple CV is biased, this observation is specific to the objective of obtaining an unbiased estimate of generalizability. Simple CV remains a valid approach for identifying optimal methods and hyperparameters. Indeed, it is utilized within the inner loop of nested CV and as the validation procedure for holdout CV for this purpose.

As the analysis suggests, a key challenge is the limited sample size available for model development. This issue is particularly pronounced in radiomics, driven by a combination of data-sharing constraints related to privacy, the labor-intensive nature of manual segmentations, and the inherent rarity of certain diseases. Such limitations may extend beyond radiomics to other domains where machine learning is applied, particularly those involving high-dimensional data. Consequently, the findings of this study may be generalizable to other fields, although radiomic datasets are distinct in their high proportion of redundant, highly correlated features resulting from preprocessing filters^38^.

Based on these findings, it is recommended that the use of simple k-fold CV be avoided in radiomic research when an independent test set is unavailable. Holdout CV with refitting provides relatively unbiased estimates across the evaluated metrics and may be considered a robust methodology. However, when an independent test set is available, nested CV with a simple ensemble yields validation estimates that are comparably unbiased as holdout CV, while generally achieving superior predictive performance. Furthermore, the results indicate that increasing the number of repetitions in nested CV helps reduce estimation variance, whereas increasing the number of outer folds does not yield a significant improvement in stability. Consequently, nested (5 × 10)-CV with a simple ensemble should be adopted as the preferred validation framework for radiomic datasets.

While this study contributes to the limited body of research examining model creation strategies within nested CV, further exploration of alternative approaches is warranted. Recent work has demonstrated the statistical equivalence of many top-performing radiomics models^38^. Given that the observed performance loss in ensembling may be attributable to the use of models sharing identical configurations, actively selecting models with distinct configurations yet comparable performance could represent a viable alternative.

Furthermore, alternative validation schemes, such as bootstrapping and leave-one-out CV, were not explored in this study due to their prohibitive computational cost and warrant investigation in future research. Given the aim of radiomics to identify biomarkers, it could be crucial to understand the extent to which different validation schemes influence feature selection and feature importance^25,39,40^.

This study has several limitations. Only three metrics were considered, however, in clinical practice, other metrics, such as specificity or sensitivity, are often of importance. Furthermore, the amount of overestimation can be substantially influenced by the number of hyperparameters. This study explored a limited set of feature selection methods and classifiers, which may underestimate the true extent of bias. Yet, given that many radiomics studies also evaluate a restricted number of configurations, the present findings could remain relevant to current practice. Finally, this study did not consider external datasets, which may exhibit different data distributions.

In conclusion, this study quantitatively evaluated the optimistic bias of several validation schemes using a large collection of radiomic data. Consistent with previous research on high-dimensional data, the findings confirmed that substantial overestimation can occur with simple k-fold cross-validation, particularly when influenced by small sample sizes. The results indicated that repeated holdout CV and nested CV reduced the overestimation and are therefore recommended for radiomics studies. Additionally, the study reinforces the conclusion that results from low-dimensional datasets do not always transfer to radiomic datasets.

## Supplementary Information

Below is the link to the electronic supplementary material.

Supplementary Material 1.

## Data Availability

The code generated and/or analyzed during the current study are available in the GitHub repository at https://github.com/aydindemircioglu/radOverzealous. The radiomic datasets can be accessed via the radMLBench repository at https://github.com/aydindemircioglu/radMLBench. The UCI datasets can be can be accessed via https://figshare.com/articles/dataset/Nested_cross_validation_is_overzelous/3457238. More information on each be found in Table 1 and the Supplementary Materials. The code and results of this study can additionally be accessed via zenodo at https://zenodo.org/records/20649231 (10.5281/zenodo.20649230).
