# Supplementary material for: Measuring the optimistic bias of cross-validation in radiomics
Source: Sci Rep. 2026 Jul 20;16:22686. doi: 10.1038/s41598-026-62792-w (PMC13385755; doi:10.1038/s41598-026-62792-w)
Supplement: Supplementary file 1 — Supplementary Material 1. [file 41598_2026_62792_MOESM1_ESM.docx]

## **Supplementary Materials**

**Measuring the Optimistic Bias of Cross-Validation in Radiomics**

**Table S1** Description of the radiomic datasets used in this study and their download URL.

| **Dataset** | **Modality** | **Short description** | **Download URL** |
| --- | --- | --- | --- |
| Ahn2021 | MRI | Pretreatment T1-weighted, contrast-enhanced T1-weighted, and T2-weighted MRI of patients with glioblastoma. | https://data.mendeley.com/public-api/zip/9mpy8mc6hn/download/1 |
| Arita2018 | MRI | Presurgical T2-weigthed MRI of patients with lower-grade glioma (WHO grade II-III). | https://www.nature.com/articles/s41598-018-30273-4#Sec18 |
| BraTS-2021 | MRI | T1-weighted, post-contrast T1-weighted, T2-weighted, T2  fluid attenuated inversion recovery (FLAIR) MRI of patients with glioblastoma. | https://radiomics.uk/ |
| Dai2023 | CT | Pretreatment contrast-enhanced abdominal CT of patients with gastrointestinal malignancies. | https://dfzljdn9uc3pi.cloudfront.net/2023/16230/1/raw_data.zip |
| Deng2023 | MRI | Pretreatment T1-weighted, T2-weighted, and contrast-enhanced T1-weighted MRI of patients with NSCLC and brain metastases. | https://github.com/BboyT/BM_NSCLC_subpathology/data |
| Dong2022 | CT | Pretreatment contrast-enhanced CT of patients with pulmonary tuberculosis or primary lung adenocarcinoma. | https://peerj.com/articles/14127/#supplemental-information |
| Head-Neck-Radiomics-HN1 | CT | Pretreatment CT of patients with head-and-neck squamous cell carcinoma. | https://radiomics.uk/ |
| Hosny2018A | CT | CT with or without intravenous contrast CT of patients with stage I-IIIb NSCLC treated with radiation therapy (HarvardRT subset). | https://github.com/modelhub-ai/deep-prognosis |
| Hosny2018B | CT | CT with or without intravenous contrast CT of patients with stage I-IIIb NSCLC treated with radiation therapy (Maastro subset). | https://github.com/modelhub-ai/deep-prognosis |
| Hosny2018C | CT | CT with or without intravenous contrast CT of patients with stage I-IIIb NSCLC treated with radiation therapy (Moffitt subset). | https://github.com/modelhub-ai/deep-prognosis |
| Huang2023 | CT | Unenhanced and dual-phase contrast-enhanced CT (arterial and venous phase) in patients with cystic renal masses. | https://journals.plos.org/plosone/article?id=10.1371/journal.pone.0292110#sec019 |
| Hunter2023 | CT | CT of patients with solid lung nodules. | https://data.mendeley.com/datasets/rxn95mp24d/1 |
| ISPY1 | MRI | DCE-MRI of patients with stage 2 or 3 breast cancer receiving neoadjuvant chemotherapy. | https://www.cancerimagingarchive.net/analysis-result/ispy1-tumor-seg-radiomics/ |
| Keek2020 | CT | Contrast-enhanced CT of therapy-naïve patients with head-and-neck squamous cell carcinoma. | https://github.com/SebastianSanduleanu/Peritumoral-HN-Radiomics |
| LGG-1p19qDeletion | MRI | Pretreatment post-contrast T1-weighted and T2-weighted MRI of patients with low-grade glioma. | https://radiomics.uk/ |
| LNDb | CT | CT of patients with lung cancer. | https://radiomics.uk/ |
| NSCLC-Radiogenomics | PET/CT | Pretreatment CT and PET/CT of patients with early-stage NSCLC. | https://radiomics.uk/ |
| PI-CAI | MRI | T2-weighted and diffusion-weighted MRI in biopsy-naïve patients. | https://radiomics.uk/ |
| Petrillo2023 | MRI | T2-weighted turbo spin-echo and dynamic contrast-enhanced MRI in patients with breast cancer. | https://zenodo.org/records/8392800 |
| Prostate-MRI-US-Biopsy | MRI | Pretreatment T2-weighted, perfusion-weighted, and diffusion-weighted MRI of patients with prostate cancer. | https://radiomics.uk/ |
| Sasaki2019 | MRI | Pretreatment T1-weighted, T2-weighted and gadolinium-enhanced MRI of patients with glioblastoma. | https://www.nature.com/articles/s41598-019-50849-y#Sec11 |
| Song2020 | MRI | T2-weighted MRI, diffusion-weighted imaging, and apparent diffusion coefficient maps in patients with prostate cancer. | https://github.com/salan668/FAE/tree/master/Feature |
| UCSF-PDGM | MRI | Pretreatment 3D T2-weighted, T2-weighted FLAIR, susceptibility weighted, diffusion-weighted, pre- and postcontrast T1-weighted MRI of patients with diffuse glioma with WHO grade II-IV. | https://radiomics.uk/ |
| UPENN-GBM | MRI | Pretreatment T1-weighted, post-contrast T1-weighted, T2-weighted and T2 fluid attenuated inversion recovery (FLAIR) MRI of patients with glioblastoma. | https://radiomics.uk/ |
| Veeraraghavan2020 | CT | Pretreatment contrast-enhanced CT in patients with endometrial cancer. | https://github.com/The-Veeraraghavan-Lab/SciRepEndometrial2020 |
| WORC-Desmoid | MRI | Pretreatment T1-weighted MRI of patients with primary or recurrent desmoid-type fibromatosis. | https://radiomics.uk/ |
| WORC-GIST | CT | Pretreatment contrast-enhanced venous-phase CT of patients with primary GIST or intra-abdominal tumors resembling GIST. | https://radiomics.uk/ |
| WORC-Lipo | MRI | Pretreatment T1-weighted MRI in patients with soft-tissue liposarcoma or lipoma. | https://radiomics.uk/ |
| WORC-Liver | MRI | T2-weighted MRI in patients with liver cancer. | https://radiomics.uk/ |
| Zhang2023 | CT | Pretreatment CT in patients with sub-centimeter subsolid pulmonary nodules. | https://peerj.com/articles/14559/#supplemental-information |
| Zhang2024A | PET/CT | Pretreatment 18F-FDG PET/CT in patients with NSCLC with stage III and a single tumor lesion exceeding 1 cm. | https://journals.plos.org/plosone/article?id=10.1371/journal.pone.0300170#sec017 |
| Zhang2024B | CT | Pretreatment, contrast-enhanced abdominal CT in patients with gastric cancer. | https://peerj.com/articles/17111/#supplemental-information |

**Table S2** Search space of the hyperparameters considered in this study for the radiomics datasets.

| **Hyperparameter** | **Possible values** |
| --- | --- |
| Feature selection method | LASSO, MRMRe, ET |
| - Number of features to retain | 1, 2, 4, … 32 |
| Classifier | NB, LR, RF, SVM |
| - For LR and SVM: | Regularization parameter C 2^-7^, 2^-5^, 2^-3^, 2^-1^, 2^0^, 2^1^, 2^3^, 2^5^, 2^7^ |
| - For RF | Maximum depth 3, 5, 7; number of trees: 50, 100, 250 |

The hyperparameters that were considered during the grid search. Overall, 3*6*(1+9+9+9) = 504 configurations were tested during the CV. ET: Extra trees; LASSO: Least Absolute Shrinkage and Selection Operator; LR: Logistic regression; MRMRe: Minimum Redundancy Maximum Relevance ensemble; NB: Naive Bayes; RF: Random forest; SVM: Support vector machine

**Table S3** UCI datasets used in this study.

| **Dataset** | **Instances** | **Features** | **Dimensionality** | **Balance** | **Source** |
| --- | --- | --- | --- | --- | --- |
| abalone | 4177 | 8 | 0,002 | 32 | <https://doi.org/10.24432/C55C7W> |
| acute-inflammation | 120 | 6 | 0,05 | 49 | <https://doi.org/10.24432/C5V59S> |
| acute-nephritis | 120 | 6 | 0,05 | 42 | <https://doi.org/10.24432/C5V59S> |
| adult | 5000 | 14 | 0,003 | 24 | <https://doi.org/10.24432/C5XW20> |
| annealing | 798 | 31 | 0,039 | 19 | <https://doi.org/10.24432/C5RW2F> |
| arrhythmia | 452 | 262 | 0,58 | 31 | <https://doi.org/10.24432/C5BS32> |
| audiology-std | 171 | 59 | 0,345 | 37 | <https://doi.org/10.24432/C5TP4R> |
| balance-scale | 625 | 4 | 0,006 | 46 | <https://doi.org/10.24432/C5488X> |
| bank | 4521 | 16 | 0,004 | 12 | <https://doi.org/10.24432/C5K306> |
| blood | 748 | 4 | 0,005 | 24 | <https://doi.org/10.24432/C5GS39> |
| breast-cancer | 286 | 9 | 0,031 | 30 | <https://doi.org/10.24432/C51P4M> |
| breast-cancer-wisc | 699 | 9 | 0,013 | 34 | <https://doi.org/10.24432/C5HP4Z> |
| breast-cancer-wisc-diag | 569 | 30 | 0,053 | 37 | <https://doi.org/10.24432/C5DW2B> |
| breast-cancer-wisc-prog | 198 | 33 | 0,167 | 24 | <https://doi.org/10.24432/C5GK50> |
| breast-tissue | 106 | 9 | 0,085 | 50 | <https://doi.org/10.24432/C5P31H> |
| car | 1728 | 6 | 0,003 | 74 | <https://doi.org/10.24432/C5JP48> |
| cardiotocography-10clases | 2126 | 21 | 0,01 | 61 | <https://doi.org/10.24432/C51S4N> |
| cardiotocography-3clases | 2126 | 21 | 0,01 | 14 | <https://doi.org/10.24432/C51S4N> |
| chess-krvk | 5000 | 6 | 0,001 | 46 | <https://doi.org/10.24432/C57W2S> |
| chess-krvkp | 3196 | 36 | 0,011 | 52 | <https://doi.org/10.24432/C5DK5C> |
| congressional-voting | 435 | 16 | 0,037 | 39 | <https://doi.org/10.24432/C5C01P> |
| conn-bench-sonar-mines-rocks | 208 | 60 | 0,288 | 47 | <https://doi.org/10.24432/C5T01Q> |
| conn-bench-vowel-deterding | 990 | 11 | 0,011 | 45 | <https://doi.org/10.24432/C58P4S> |
| connect-4 | 5000 | 42 | 0,008 | 25 | <https://doi.org/10.24432/C59P43> |
| contrac | 1473 | 9 | 0,006 | 23 | <https://doi.org/10.24432/C59W2D> |
| credit-approval | 690 | 15 | 0,022 | 56 | <https://doi.org/10.24432/C5FS30> |
| cylinder-bands | 512 | 35 | 0,068 | 61 | <https://doi.org/10.24432/C50C7B> |
| dermatology | 366 | 34 | 0,093 | 36 | <https://doi.org/10.24432/C5FK5P> |
| echocardiogram | 131 | 10 | 0,076 | 33 | <https://doi.org/10.24432/C5QW24> |
| ecoli | 336 | 7 | 0,021 | 35 | <https://doi.org/10.24432/C5388M> |
| energy-y1 | 768 | 8 | 0,01 | 18 | <https://doi.org/10.24432/C51307> |
| energy-y2 | 768 | 8 | 0,01 | 26 | <https://doi.org/10.24432/C51307> |
| flags | 194 | 28 | 0,144 | 51 | <https://doi.org/10.24432/C52C7Z> |
| glass | 214 | 9 | 0,042 | 55 | <https://doi.org/10.24432/C5WW2P> |
| haberman-survival | 306 | 3 | 0,01 | 26 | <https://doi.org/10.24432/C5XK51> |
| hayes-roth | 160 | 3 | 0,019 | 40 | <https://doi.org/10.24432/C5501T> |
| heart-cleveland | 303 | 13 | 0,043 | 30 | <https://doi.org/10.24432/C52P4X> |
| heart-hungarian | 294 | 12 | 0,041 | 36 | <https://doi.org/10.24432/C52P4X> |
| heart-switzerland | 123 | 12 | 0,098 | 63 | <https://doi.org/10.24432/C52P4X> |
| heart-va | 200 | 12 | 0,06 | 49 | <https://doi.org/10.24432/C52P4X> |
| hepatitis | 155 | 19 | 0,123 | 79 | <https://doi.org/10.24432/C5Q59J> |
| hill-valley | 1212 | 100 | 0,083 | 50 | <https://doi.org/10.24432/C5JC8P> |
| horse-colic | 368 | 25 | 0,068 | 37 | <https://doi.org/10.24432/C58W23> |
| ilpd-indian-liver | 583 | 9 | 0,015 | 29 | <https://doi.org/10.24432/C5D02C> |
| image-segmentation | 2310 | 18 | 0,008 | 43 | <https://doi.org/10.24432/C5GP4N> |
| ionosphere | 351 | 33 | 0,094 | 64 | <https://doi.org/10.24432/C5W01B> |
| iris | 150 | 4 | 0,027 | 33 | <https://doi.org/10.24432/C56C76> |
| led-display | 1000 | 7 | 0,007 | 50 | <https://doi.org/10.24432/C5FG61> |
| letter | 5000 | 16 | 0,003 | 50 | <https://doi.org/10.24432/C5ZP40> |
| libras | 360 | 90 | 0,25 | 47 | <https://doi.org/10.24432/C5GC82> |
| low-res-spect | 531 | 100 | 0,188 | 73 | <https://doi.org/10.24432/C5B02R> |
| lymphography | 148 | 18 | 0,122 | 57 | <https://doi.org/10.24432/C54598> |
| magic | 5000 | 10 | 0,002 | 35 | <https://doi.org/10.24432/C52C8B> |
| mammographic | 961 | 5 | 0,005 | 46 | <https://doi.org/10.24432/C53K6Z> |
| miniboone | 5000 | 50 | 0,01 | 72 | <https://doi.org/10.24432/C5QC87> |
| molec-biol-promoter | 106 | 57 | 0,538 | 50 | <https://doi.org/10.24432/C5S01D> |
| molec-biol-splice | 3190 | 60 | 0,019 | 24 | <https://doi.org/10.24432/C5M888> |
| monks-1 | 556 | 6 | 0,011 | 50 | <https://doi.org/10.24432/C5R30R> |
| monks-2 | 601 | 6 | 0,01 | 34 | <https://doi.org/10.24432/C5R30R> |
| monks-3 | 554 | 6 | 0,011 | 52 | <https://doi.org/10.24432/C5R30R> |
| mushroom | 5000 | 21 | 0,004 | 48 | <https://doi.org/10.24432/C5959T> |
| musk-1 | 476 | 166 | 0,349 | 43 | <https://doi.org/10.24432/C5ZK5B> |
| musk-2 | 5000 | 166 | 0,033 | 15 | <https://doi.org/10.24432/C51608> |
| nursery | 5000 | 8 | 0,002 | 33 | <https://doi.org/10.24432/C5P88W> |
| oocytes_merluccius_nucleus_4d | 1022 | 41 | 0,04 | 67 | not available on the UCI, see <https://figshare.com/articles/dataset/Nested_cross_validation_is_overzelous/3457238> |
| oocytes_merluccius_states_2f | 1022 | 25 | 0,024 | 6 | not available on the UCI, see <https://figshare.com/articles/dataset/Nested_cross_validation_is_overzelous/3457238> |
| oocytes_trisopterus_nucleus_2f | 912 | 25 | 0,027 | 58 | not available on the UCI, see <https://figshare.com/articles/dataset/Nested_cross_validation_is_overzelous/3457238> |
| optical | 3823 | 62 | 0,016 | 50 | <https://doi.org/10.24432/C50P49> |
| ozone | 2536 | 72 | 0,028 | 3 | <https://doi.org/10.24432/C5NG6W> |
| page-blocks | 5000 | 10 | 0,002 | 8 | <https://doi.org/10.24432/C5J590> |
| parkinsons | 195 | 22 | 0,113 | 75 | <https://doi.org/10.24432/C59C74> |
| pendigits | 5000 | 16 | 0,003 | 50 | <https://doi.org/10.24432/C5MG6K> |
| pima | 768 | 8 | 0,01 | 35 | not available on the UCI, see <https://github.com/npradaschnor/Pima-Indians-Diabetes-Dataset> |
| pittsburg-bridges-TYPE | 105 | 7 | 0,067 | 32 | <https://doi.org/10.24432/C5RP5H> |
| planning | 182 | 12 | 0,066 | 29 | <https://doi.org/10.24432/C5T023> |
| plant-margin | 1600 | 64 | 0,04 | 50 | <https://doi.org/10.24432/C5HS40> |
| plant-shape | 1600 | 64 | 0,04 | 50 | <https://doi.org/10.24432/C5HS40> |
| plant-texture | 1599 | 64 | 0,04 | 50 | <https://doi.org/10.24432/C5HS40> |
| primary-tumor | 330 | 17 | 0,052 | 30 | <https://doi.org/10.24432/C5WK5Q> |
| ringnorm | 5000 | 20 | 0,004 | 50 | not available on the UCI, see <https://www.cs.toronto.edu/~delve/data/datasets.html> |
| seeds | 210 | 7 | 0,033 | 33 | <https://doi.org/10.24432/C5H30K> |
| semeion | 1593 | 256 | 0,161 | 50 | <https://doi.org/10.24432/C5SC8V> |
| soybean | 683 | 35 | 0,051 | 58 | <https://doi.org/10.24432/C5JG6Z> |
| spambase | 4601 | 57 | 0,012 | 39 | <https://doi.org/10.24432/C53G6X> |
| spect | 265 | 22 | 0,083 | 42 | <https://doi.org/10.24432/C5P304> |
| spectf | 267 | 44 | 0,165 | 79 | <https://doi.org/10.24432/C5N015> |
| statlog-australian-credit | 690 | 14 | 0,02 | 68 | <https://doi.org/10.24432/C59012> |
| statlog-german-credit | 1000 | 24 | 0,024 | 30 | <https://doi.org/10.24432/C5NC77> |
| statlog-heart | 270 | 13 | 0,048 | 44 | <https://doi.org/10.24432/C57303> |
| statlog-image | 2310 | 18 | 0,008 | 43 | <https://doi.org/10.24432/C5P01G> |
| statlog-landsat | 5000 | 36 | 0,007 | 44 | <https://doi.org/10.24432/C55887> |
| statlog-shuttle | 5000 | 9 | 0,002 | 15 | <https://doi.org/10.24432/C5WS31> |
| statlog-vehicle | 846 | 18 | 0,021 | 49 | <https://doi.org/10.24432/C5HG6N> |
| steel-plates | 1941 | 27 | 0,014 | 34 | <https://doi.org/10.24432/C5J88N> |
| synthetic-control | 600 | 60 | 0,1 | 50 | <https://doi.org/10.24432/C59G75> |
| teaching | 151 | 5 | 0,033 | 33 | <https://doi.org/10.24432/C55P6M> |
| thyroid | 3772 | 21 | 0,006 | 5 | <https://doi.org/10.24432/C5D010> |
| tic-tac-toe | 958 | 9 | 0,009 | 65 | <https://doi.org/10.24432/C5688J> |
| titanic | 2201 | 3 | 0,001 | 32 | not available on the UCI, see <https://www.openml.org/search?type=data&sort=runs&id=40945&status=active> |
| twonorm | 5000 | 20 | 0,004 | 50 | not available on the UCI, see <https://www.cs.toronto.edu/~delve/data/datasets.html> |
| vertebral-column-2clases | 310 | 6 | 0,019 | 32 | <https://doi.org/10.24432/C5K89B> |
| vertebral-column-3clases | 310 | 6 | 0,019 | 32 | <https://doi.org/10.24432/C5K89B> |
| wall-following | 5000 | 24 | 0,005 | 21 | <https://doi.org/10.24432/C57C8W> |
| waveform | 5000 | 21 | 0,004 | 33 | <https://doi.org/10.24432/C5CS3C> |
| waveform-noise | 5000 | 40 | 0,008 | 33 | <https://doi.org/10.24432/C56014> |
| wine | 178 | 13 | 0,073 | 40 | <https://doi.org/10.24432/C5PC7J> |
| wine-quality-red | 1599 | 11 | 0,007 | 44 | <https://doi.org/10.24432/C56S3T> |
| wine-quality-white | 4898 | 11 | 0,002 | 52 | <https://doi.org/10.24432/C56S3T> |
| yeast | 1484 | 8 | 0,005 | 45 | <https://doi.org/10.24432/C5KG68> |
| zoo | 101 | 16 | 0,158 | 41 | <https://doi.org/10.24432/C5R59V> |

**Table S4** Search space of the hyperparameters considered in this study for the UCI datasets.

| **Hyperparameter** | **Possible values** |
| --- | --- |
| Classifier | NB, LR, RF, SVM |
| - For LR and SVM: | Regularization parameter C 2^-7^, 2^-5^, 2^-3^, 2^-1^, 2^0^, 2^1^, 2^3^, 2^5^, 2^7^ |
| - For RF | Maximum depth 3, 5, 7; number of trees: 50, 100, 250 |

The hyperparameters that were considered during the grid search. Overall, (1+9+9+9) = 28 configurations were tested during the CV. ET: Extra trees; LASSO: Least Absolute Shrinkage and Selection Operator; LR: Logistic regression; MRMRe: Minimum Redundancy Maximum Relevance ensemble; NB: Naive Bayes; RF: Random forest; SVM: Support vector machine

**Figure S1** Association between the amount of overestimation in F1 and dataset characteristics

**
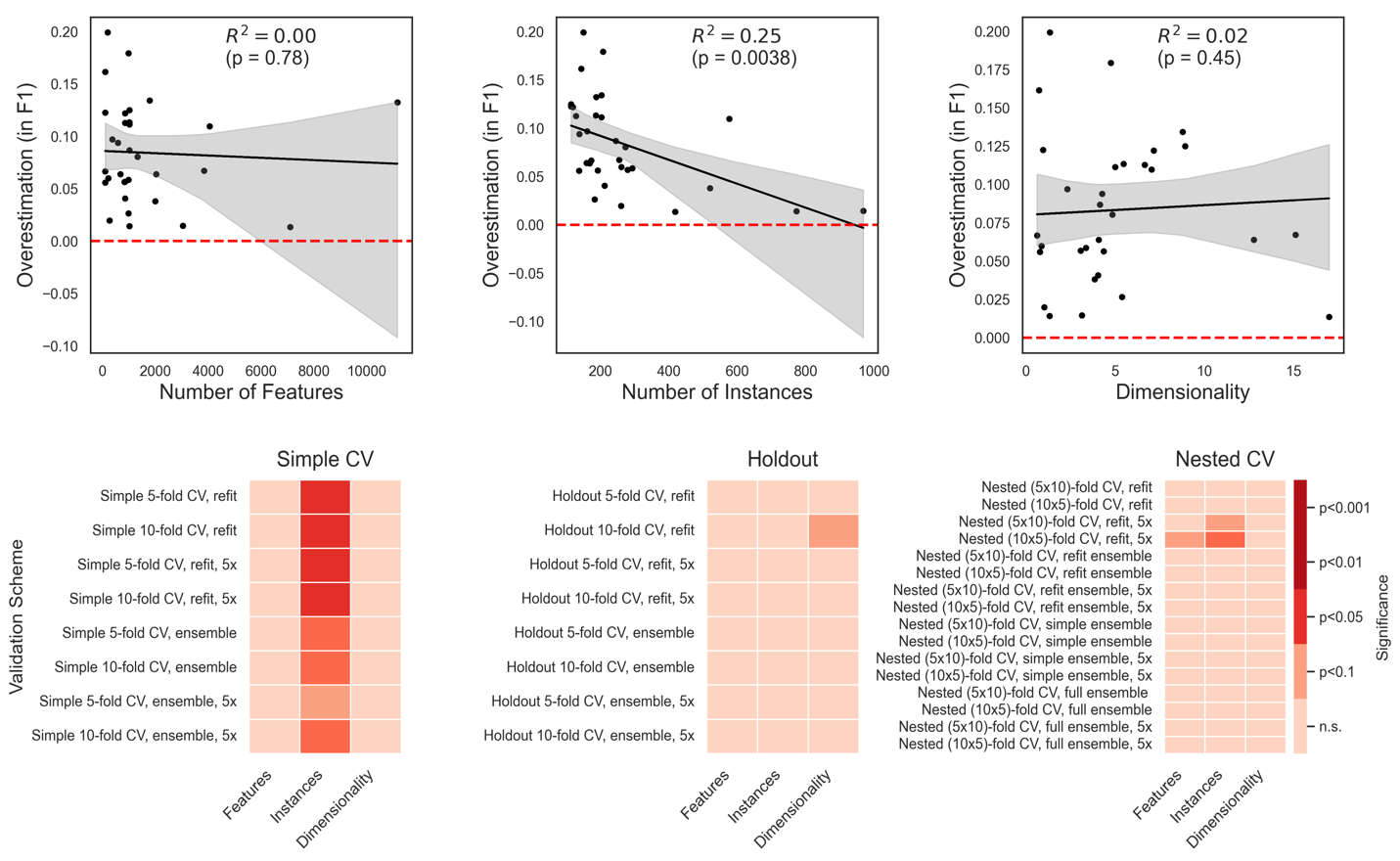
**

Top row: Scatter plots for simple 5-fold CV demonstrating a significant association between the amount of overestimation in F1 scores and the number of instances. Bottom row: Heatmaps summarizing the statistical significance (p-values) of the association between overestimation and dataset characteristics across all validation schemes. The three scatter plots shown in the top row correspond to the first row (‘Simple 5-fold CV, refit’) of the ‘Simple CV’ heatmap. For improved clarity, two datasets, Brancato2023 and UPENN-GBM, both of which have a dimensionality greater than 40, were excluded from the plots involving dimensionality.

**Figure S2** Graphical boxplot of the overestimation in MCC for two datasets

**
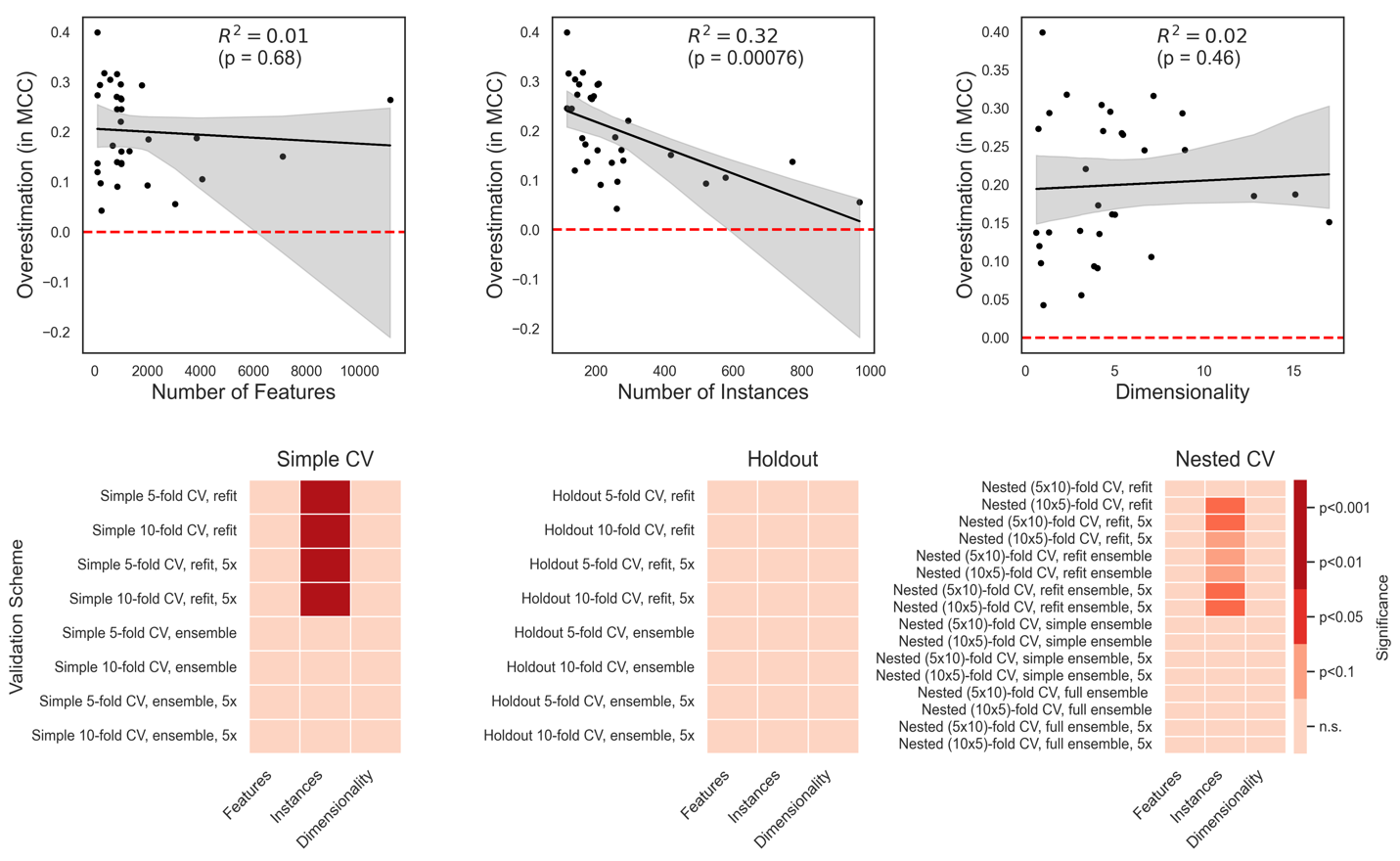
**

Top row: Scatter plots for simple 5-fold CV demonstrating a significant association between the amount of overestimation in MCC and the number of instances. Bottom row: Heatmaps summarizing the statistical significance (p-values) of the association between overestimation and dataset characteristics across all validation schemes. The three scatter plots shown in the top row correspond to the first row (‘Simple 5-fold CV, refit’) of the ‘Simple CV’ heatmap. For improved clarity, two datasets, Brancato2023 and UPENN-GBM, both of which have a dimensionality greater than 40, were excluded from the plots involving dimensionality.

**Figure S3** Graphical boxplot of the standard deviation of the overestimation amount in F1-score

**
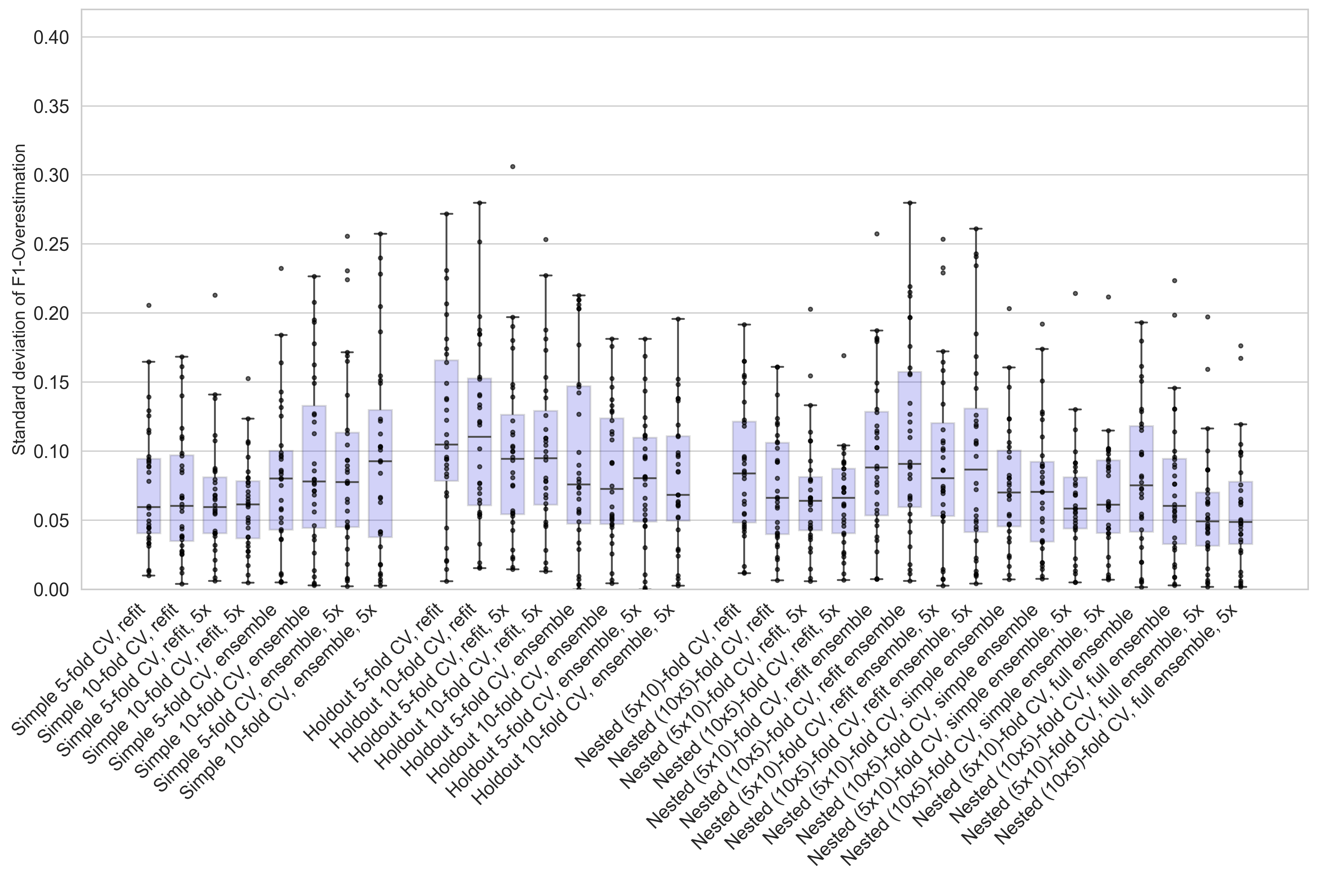
**

For each dataset and validation scheme, the standard deviation across the 10 repeats were averaged and plotted.

**Figure S4** Graphical boxplot of the standard deviation of the overestimation amount in MCC

**
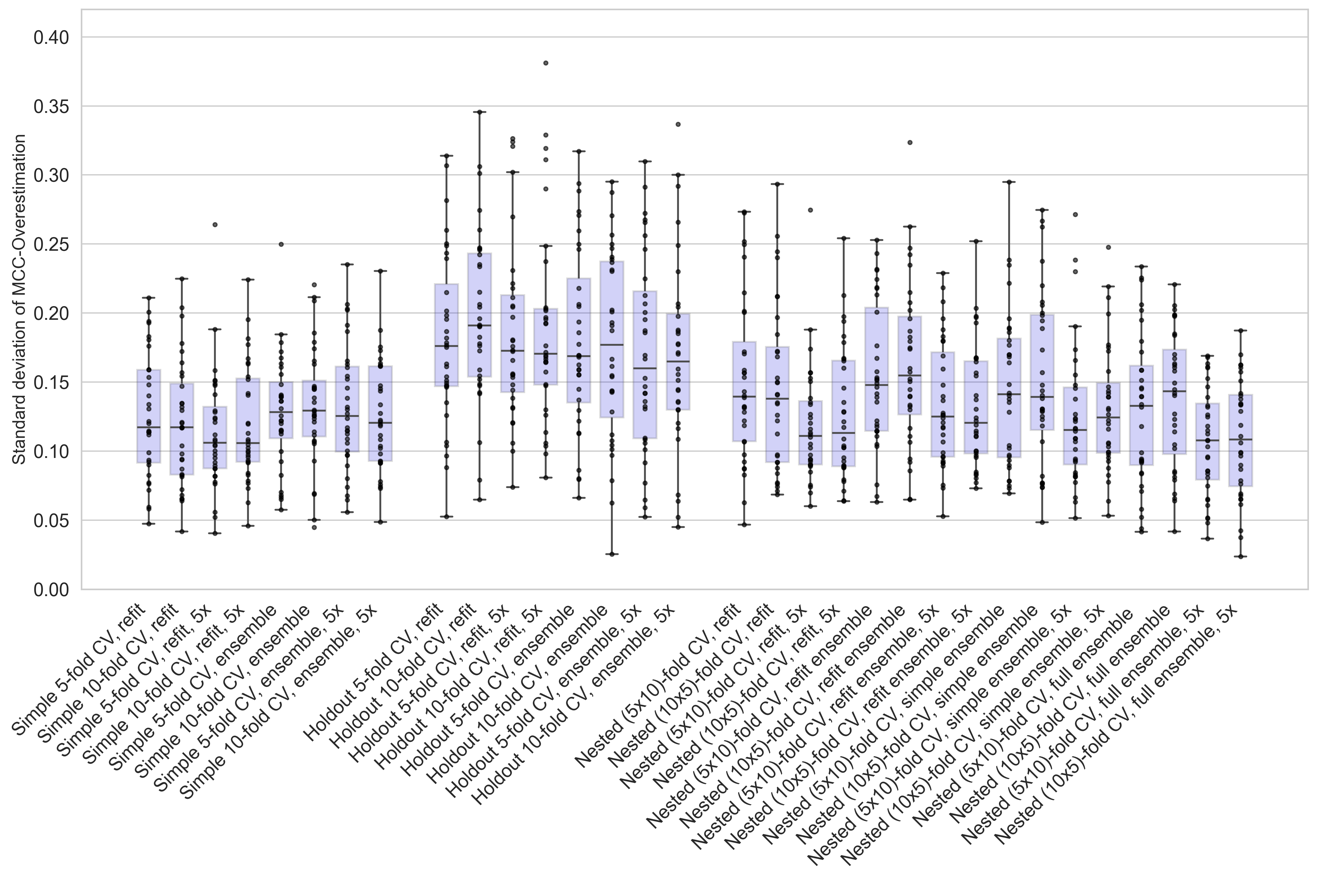
**

For each dataset and validation scheme, the standard deviation across the 10 repeats were averaged and plotted.

**Figure S5** Graphical plot of the computation times of the validation schemes for the radiomic datasets across all metrics.


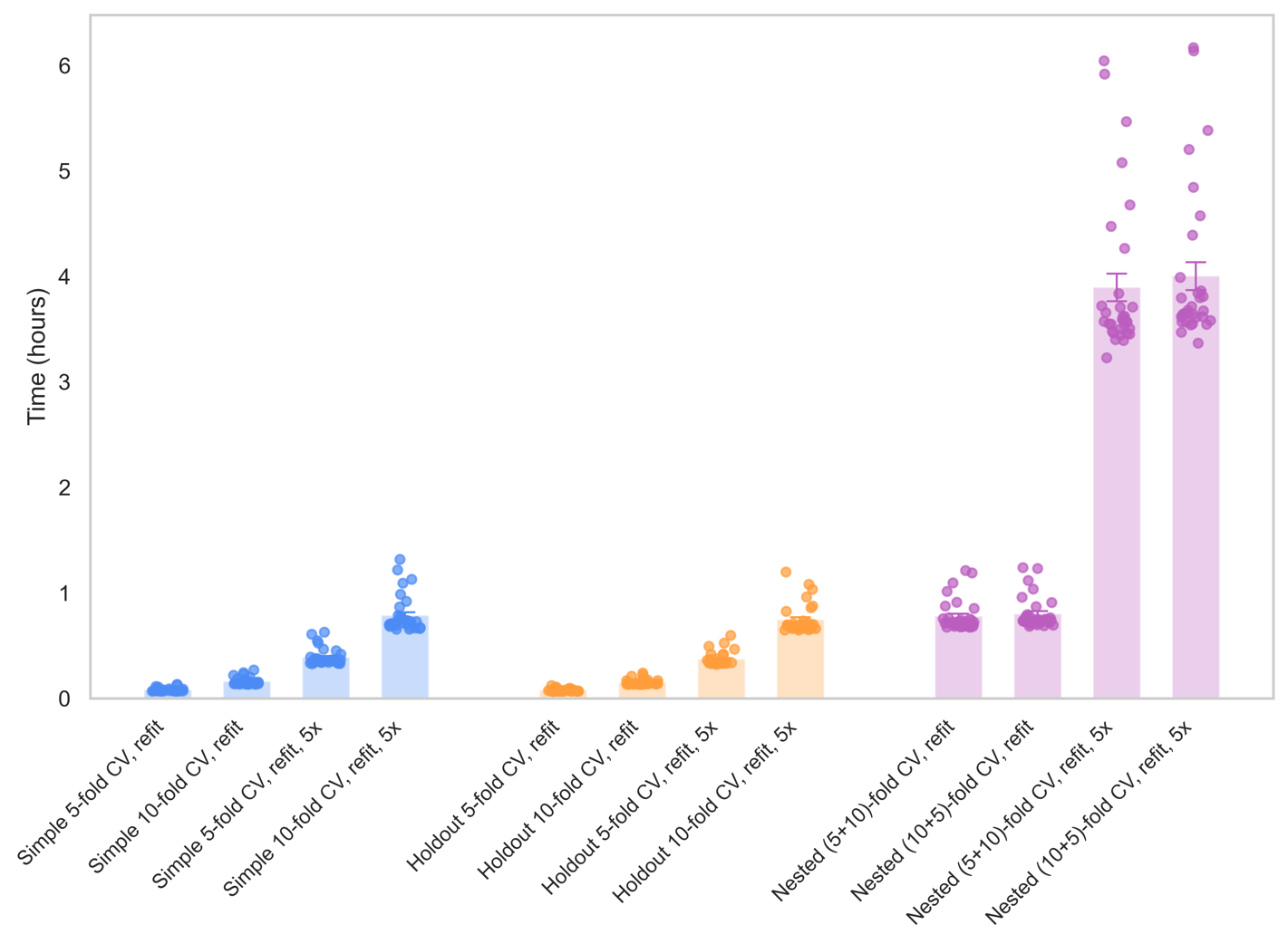


Scatter and bar plot of the computation times of the validation schemes employed. Each dot denotes the time taken of one repeat over all datasets across all three metrics.

**Table S5** Overview of the performance estimates in AUC for the UCI datasets.

| **CV** | **Folds** | **Repeats** | **Model** | **AUC-Int** | **AUC-Test** | **Optimistic Bias** | **Relative Performance** |
| --- | --- | --- | --- | --- | --- | --- | --- |
| Simple CV | 5 | 1 | Refit | 0.894 ± 0.114 | 0.89 ± 0.126 | 0.004 ± 0.023 | -0.006 ± 0.008 |
|  |  |  | Ensemble | 0.894 ± 0.114 | 0.886 ± 0.126 | 0.008 ± 0.021 | -0.01 ± 0.011 |
|  |  | 5 | Refit | 0.891 ± 0.117 | 0.89 ± 0.126 | 0.001 ± 0.02 | -0.005 ± 0.008 |
|  |  |  | Ensemble | 0.891 ± 0.117 | 0.886 ± 0.126 | 0.005 ± 0.018 | -0.009 ± 0.01 |
|  | 10 | 1 | Refit | 0.895 ± 0.115 | 0.889 ± 0.127 | 0.006 ± 0.024 | -0.006 ± 0.009 |
|  |  |  | Ensemble | 0.895 ± 0.115 | 0.887 ± 0.128 | 0.008 ± 0.024 | -0.008 ± 0.01 |
|  |  | 5 | Refit | 0.894 ± 0.116 | 0.89 ± 0.126 | 0.004 ± 0.02 | -0.005 ± 0.008 |
|  |  |  | Ensemble | 0.894 ± 0.116 | 0.889 ± 0.126 | 0.006 ± 0.02 | -0.007 ± 0.009 |
| Holdout CV | 5 | 1 | Refit | 0.881 ± 0.129 | 0.887 ± 0.126 | -0.007 ± 0.019 | -0.008 ± 0.013 |
|  |  |  | Ensemble | 0.876 ± 0.13 | 0.874 ± 0.132 | 0.001 ± 0.014 | -0.021 ± 0.022 |
|  |  | 5 | Refit | 0.881 ± 0.128 | 0.889 ± 0.126 | -0.008 ± 0.021 | -0.006 ± 0.009 |
|  |  |  | Ensemble | 0.876 ± 0.13 | 0.876 ± 0.131 | 0.001 ± 0.018 | -0.02 ± 0.022 |
|  | 10 | 1 | Refit | 0.881 ± 0.126 | 0.889 ± 0.126 | -0.007 ± 0.018 | -0.007 ± 0.01 |
|  |  |  | Ensemble | 0.879 ± 0.127 | 0.878 ± 0.13 | 0.001 ± 0.015 | -0.017 ± 0.018 |
|  |  | 5 | Refit | 0.882 ± 0.127 | 0.889 ± 0.127 | -0.007 ± 0.021 | -0.006 ± 0.01 |
|  |  |  | Ensemble | 0.878 ± 0.131 | 0.878 ± 0.131 | 0.0 ± 0.019 | -0.018 ± 0.018 |
| Nested CV | 5+10 | 1 | Refit | 0.885 ± 0.127 | 0.89 ± 0.126 | -0.005 ± 0.012 | -0.006 ± 0.007 |
|  |  |  | Refit ensemble | 0.885 ± 0.127 | 0.886 ± 0.126 | -0.001 ± 0.012 | -0.01 ± 0.011 |
|  |  |  | Simple ensemble | 0.885 ± 0.127 | 0.892 ± 0.125 | -0.007 ± 0.015 | -0.003 ± 0.007 |
|  |  |  | Full ensemble | 0.882 ± 0.128 | 0.891 ± 0.126 | -0.009 ± 0.017 | -0.004 ± 0.008 |
|  |  | 5 | Refit | 0.884 ± 0.127 | 0.89 ± 0.126 | -0.006 ± 0.013 | -0.005 ± 0.008 |
|  |  |  | Refit ensemble | 0.884 ± 0.127 | 0.886 ± 0.126 | -0.002 ± 0.011 | -0.009 ± 0.01 |
|  |  |  | Simple ensemble | 0.884 ± 0.127 | 0.893 ± 0.124 | -0.009 ± 0.015 | -0.002 ± 0.006 |
|  |  |  | Full ensemble | 0.882 ± 0.128 | 0.892 ± 0.124 | -0.01 ± 0.017 | -0.003 ± 0.007 |
|  | 10+5 | 1 | Refit | 0.887 ± 0.128 | 0.889 ± 0.127 | -0.002 ± 0.01 | -0.006 ± 0.009 |
|  |  |  | Refit ensemble | 0.887 ± 0.128 | 0.887 ± 0.128 | 0.0 ± 0.01 | -0.008 ± 0.01 |
|  |  |  | Simple ensemble | 0.887 ± 0.128 | 0.893 ± 0.125 | -0.006 ± 0.011 | -0.003 ± 0.007 |
|  |  |  | Full ensemble | 0.883 ± 0.127 | 0.891 ± 0.126 | -0.008 ± 0.015 | -0.004 ± 0.008 |
|  |  | 5 | Refit | 0.887 ± 0.126 | 0.89 ± 0.126 | -0.003 ± 0.011 | -0.005 ± 0.008 |
|  |  |  | Refit ensemble | 0.887 ± 0.126 | 0.889 ± 0.126 | -0.002 ± 0.011 | -0.007 ± 0.009 |
|  |  |  | Simple ensemble | 0.887 ± 0.126 | 0.894 ± 0.124 | -0.007 ± 0.013 | -0.002 ± 0.007 |
|  |  |  | Full ensemble | 0.883 ± 0.127 | 0.892 ± 0.125 | -0.009 ± 0.016 | -0.003 ± 0.008 |

For k-fold CV, performance estimation is independent of model creation. While refit and refit ensembles for nested CV both use another internal k-fold CV for the model creation, this model uses a different split, thus the estimates are not the same as those of the k-fold CV.

**Table S6** Overview of the performance estimates in F1-score for the UCI datasets.

| **CV** | **Folds** | **Repeats** | **Model** | **F1-Int** | **F1-Test** | **Optimistic Bias** | **Relative Performance** |
| --- | --- | --- | --- | --- | --- | --- | --- |
| Simple CV | 5 | 1 | Refit | 0.805 ± 0.182 | 0.792 ± 0.191 | 0.013 ± 0.03 | -0.012 ± 0.02 |
|  |  |  | Ensemble | 0.805 ± 0.182 | 0.786 ± 0.192 | 0.019 ± 0.025 | -0.017 ± 0.017 |
|  |  | 5 | Refit | 0.801 ± 0.183 | 0.794 ± 0.19 | 0.007 ± 0.024 | -0.009 ± 0.013 |
|  |  |  | Ensemble | 0.801 ± 0.183 | 0.789 ± 0.188 | 0.011 ± 0.021 | -0.014 ± 0.014 |
|  | 10 | 1 | Refit | 0.808 ± 0.182 | 0.792 ± 0.191 | 0.016 ± 0.027 | -0.011 ± 0.015 |
|  |  |  | Ensemble | 0.808 ± 0.182 | 0.788 ± 0.196 | 0.021 ± 0.037 | -0.016 ± 0.031 |
|  |  | 5 | Refit | 0.805 ± 0.183 | 0.795 ± 0.19 | 0.01 ± 0.022 | -0.009 ± 0.012 |
|  |  |  | Ensemble | 0.805 ± 0.183 | 0.789 ± 0.195 | 0.016 ± 0.034 | -0.014 ± 0.03 |
| Holdout CV | 5 | 1 | Refit | 0.78 ± 0.194 | 0.789 ± 0.195 | -0.009 ± 0.023 | -0.014 ± 0.019 |
|  |  |  | Ensemble | 0.775 ± 0.195 | 0.773 ± 0.193 | 0.002 ± 0.023 | -0.03 ± 0.031 |
|  |  | 5 | Refit | 0.782 ± 0.191 | 0.79 ± 0.195 | -0.009 ± 0.025 | -0.013 ± 0.019 |
|  |  |  | Ensemble | 0.776 ± 0.191 | 0.776 ± 0.19 | -0.001 ± 0.017 | -0.027 ± 0.029 |
|  | 10 | 1 | Refit | 0.779 ± 0.194 | 0.79 ± 0.194 | -0.011 ± 0.027 | -0.013 ± 0.019 |
|  |  |  | Ensemble | 0.777 ± 0.194 | 0.776 ± 0.193 | 0.001 ± 0.022 | -0.027 ± 0.029 |
|  |  | 5 | Refit | 0.782 ± 0.191 | 0.791 ± 0.194 | -0.01 ± 0.024 | -0.012 ± 0.018 |
|  |  |  | Ensemble | 0.78 ± 0.192 | 0.778 ± 0.193 | 0.002 ± 0.016 | -0.026 ± 0.027 |
| Nested CV | 5+10 | 1 | Refit | 0.783 ± 0.196 | 0.792 ± 0.191 | -0.009 ± 0.024 | -0.011 ± 0.02 |
|  |  |  | Refit ensemble | 0.783 ± 0.196 | 0.786 ± 0.192 | -0.003 ± 0.016 | -0.017 ± 0.017 |
|  |  |  | Simple ensemble | 0.783 ± 0.196 | 0.794 ± 0.193 | -0.011 ± 0.02 | -0.01 ± 0.017 |
|  |  |  | Full ensemble | 0.778 ± 0.198 | 0.79 ± 0.195 | -0.012 ± 0.023 | -0.013 ± 0.024 |
|  |  | 5 | Refit | 0.784 ± 0.194 | 0.794 ± 0.19 | -0.01 ± 0.02 | -0.009 ± 0.013 |
|  |  |  | Refit ensemble | 0.784 ± 0.194 | 0.789 ± 0.188 | -0.005 ± 0.019 | -0.014 ± 0.014 |
|  |  |  | Simple ensemble | 0.784 ± 0.194 | 0.796 ± 0.192 | -0.012 ± 0.021 | -0.007 ± 0.015 |
|  |  |  | Full ensemble | 0.779 ± 0.197 | 0.793 ± 0.194 | -0.014 ± 0.022 | -0.01 ± 0.02 |
|  | 10+5 | 1 | Refit | 0.785 ± 0.198 | 0.792 ± 0.192 | -0.007 ± 0.02 | -0.011 ± 0.016 |
|  |  |  | Refit ensemble | 0.785 ± 0.198 | 0.787 ± 0.196 | -0.002 ± 0.031 | -0.016 ± 0.033 |
|  |  |  | Simple ensemble | 0.785 ± 0.198 | 0.797 ± 0.191 | -0.011 ± 0.019 | -0.007 ± 0.012 |
|  |  |  | Full ensemble | 0.776 ± 0.202 | 0.791 ± 0.195 | -0.015 ± 0.022 | -0.013 ± 0.024 |
|  |  | 5 | Refit | 0.785 ± 0.197 | 0.795 ± 0.19 | -0.01 ± 0.017 | -0.009 ± 0.012 |
|  |  |  | Refit ensemble | 0.785 ± 0.197 | 0.789 ± 0.195 | -0.004 ± 0.03 | -0.014 ± 0.031 |
|  |  |  | Simple ensemble | 0.785 ± 0.197 | 0.797 ± 0.192 | -0.012 ± 0.019 | -0.007 ± 0.015 |
|  |  |  | Full ensemble | 0.776 ± 0.2 | 0.791 ± 0.196 | -0.014 ± 0.019 | -0.013 ± 0.027 |

For k-fold CV, performance estimation is independent of model creation. While refit and refit ensembles for nested CV both use another internal k-fold CV for the model creation, this model uses a different split, thus the estimates are not the same as those of the k-fold CV.

**Table S7** Overview of the performance estimates in MCC for the UCI datasets

| **CV** | **Folds** | **Repeats** | **Model** | **MCC-Int** | **MCC-Test** | **Optimistic Bias** | **Relative Performance** |
| --- | --- | --- | --- | --- | --- | --- | --- |
| Simple CV | 5 | 1 | Refit | 0.68 ± 0.257 | 0.653 ± 0.284 | 0.027 ± 0.051 | -0.018 ± 0.019 |
|  |  |  | Ensemble | 0.68 ± 0.257 | 0.643 ± 0.284 | 0.038 ± 0.049 | -0.028 ± 0.025 |
|  |  | 5 | Refit | 0.669 ± 0.264 | 0.655 ± 0.284 | 0.014 ± 0.042 | -0.016 ± 0.02 |
|  |  |  | Ensemble | 0.669 ± 0.264 | 0.646 ± 0.281 | 0.024 ± 0.039 | -0.025 ± 0.025 |
|  | 10 | 1 | Refit | 0.685 ± 0.259 | 0.654 ± 0.282 | 0.031 ± 0.045 | -0.017 ± 0.02 |
|  |  |  | Ensemble | 0.685 ± 0.259 | 0.65 ± 0.282 | 0.035 ± 0.045 | -0.021 ± 0.022 |
|  |  | 5 | Refit | 0.677 ± 0.263 | 0.655 ± 0.284 | 0.022 ± 0.041 | -0.016 ± 0.019 |
|  |  |  | Ensemble | 0.677 ± 0.263 | 0.651 ± 0.282 | 0.026 ± 0.039 | -0.02 ± 0.021 |
| Holdout CV | 5 | 1 | Refit | 0.631 ± 0.287 | 0.648 ± 0.285 | -0.017 ± 0.04 | -0.022 ± 0.025 |
|  |  |  | Ensemble | 0.625 ± 0.285 | 0.62 ± 0.286 | 0.005 ± 0.04 | -0.051 ± 0.047 |
|  |  | 5 | Refit | 0.633 ± 0.286 | 0.653 ± 0.284 | -0.02 ± 0.042 | -0.018 ± 0.022 |
|  |  |  | Ensemble | 0.624 ± 0.286 | 0.623 ± 0.285 | 0.001 ± 0.035 | -0.048 ± 0.046 |
|  | 10 | 1 | Refit | 0.632 ± 0.29 | 0.65 ± 0.286 | -0.018 ± 0.042 | -0.021 ± 0.025 |
|  |  |  | Ensemble | 0.628 ± 0.289 | 0.626 ± 0.286 | 0.003 ± 0.036 | -0.045 ± 0.041 |
|  |  | 5 | Refit | 0.634 ± 0.284 | 0.653 ± 0.282 | -0.019 ± 0.039 | -0.018 ± 0.019 |
|  |  |  | Ensemble | 0.634 ± 0.282 | 0.632 ± 0.28 | 0.003 ± 0.032 | -0.039 ± 0.035 |
| Nested CV | 5+10 | 1 | Refit | 0.642 ± 0.283 | 0.652 ± 0.285 | -0.01 ± 0.026 | -0.018 ± 0.02 |
|  |  |  | Refit ensemble | 0.642 ± 0.283 | 0.643 ± 0.284 | -0.001 ± 0.023 | -0.028 ± 0.025 |
|  |  |  | Simple ensemble | 0.642 ± 0.283 | 0.658 ± 0.28 | -0.015 ± 0.029 | -0.013 ± 0.018 |
|  |  |  | Full ensemble | 0.636 ± 0.284 | 0.654 ± 0.28 | -0.018 ± 0.03 | -0.017 ± 0.022 |
|  |  | 5 | Refit | 0.643 ± 0.283 | 0.655 ± 0.284 | -0.013 ± 0.028 | -0.015 ± 0.02 |
|  |  |  | Refit ensemble | 0.643 ± 0.283 | 0.646 ± 0.281 | -0.003 ± 0.027 | -0.025 ± 0.025 |
|  |  |  | Simple ensemble | 0.643 ± 0.283 | 0.66 ± 0.279 | -0.018 ± 0.029 | -0.011 ± 0.014 |
|  |  |  | Full ensemble | 0.637 ± 0.284 | 0.658 ± 0.279 | -0.021 ± 0.029 | -0.012 ± 0.015 |
|  | 10+5 | 1 | Refit | 0.648 ± 0.287 | 0.654 ± 0.282 | -0.006 ± 0.027 | -0.017 ± 0.019 |
|  |  |  | Refit ensemble | 0.648 ± 0.287 | 0.65 ± 0.282 | -0.002 ± 0.027 | -0.021 ± 0.021 |
|  |  |  | Simple ensemble | 0.648 ± 0.287 | 0.661 ± 0.279 | -0.013 ± 0.03 | -0.01 ± 0.013 |
|  |  |  | Full ensemble | 0.637 ± 0.287 | 0.656 ± 0.28 | -0.019 ± 0.032 | -0.014 ± 0.017 |
|  |  | 5 | Refit | 0.649 ± 0.284 | 0.655 ± 0.284 | -0.006 ± 0.023 | -0.015 ± 0.019 |
|  |  |  | Refit ensemble | 0.649 ± 0.284 | 0.651 ± 0.282 | -0.002 ± 0.023 | -0.02 ± 0.021 |
|  |  |  | Simple ensemble | 0.649 ± 0.284 | 0.661 ± 0.281 | -0.012 ± 0.027 | -0.01 ± 0.016 |
|  |  |  | Full ensemble | 0.638 ± 0.284 | 0.657 ± 0.28 | -0.018 ± 0.03 | -0.014 ± 0.017 |

For k-fold CV, performance estimation is independent of model creation. While refit and refit ensembles for nested CV both use another internal k-fold CV for the model creation, this model uses a different split, thus the estimates are not the same as those of the k-fold CV.

**Figure S6** Scatter plot of the AUC comparing validation and test estimates (amount of overestimation) for the UCI datasets.


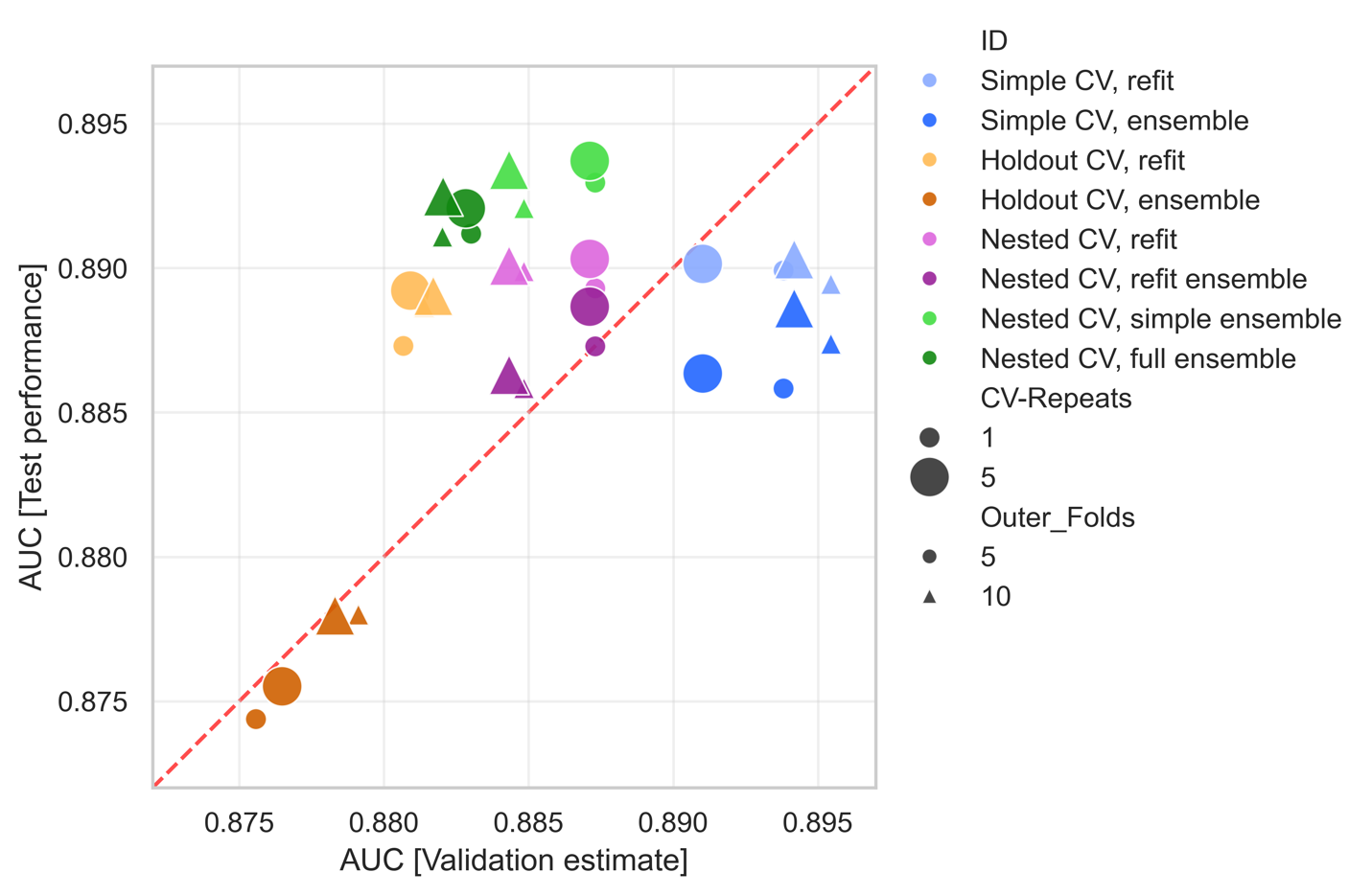

CV schemes below the dashed line indicate overestimation, while those above suggest underestimation of the true performance. Schemes positioned higher in the plot demonstrate better performance on the test set, whereas those positioned lower exhibit poorer performance.

**Figure S7** Scatter plot of the F1 scores comparing validation and test estimates (amount of overestimation) for the UCI datasets.


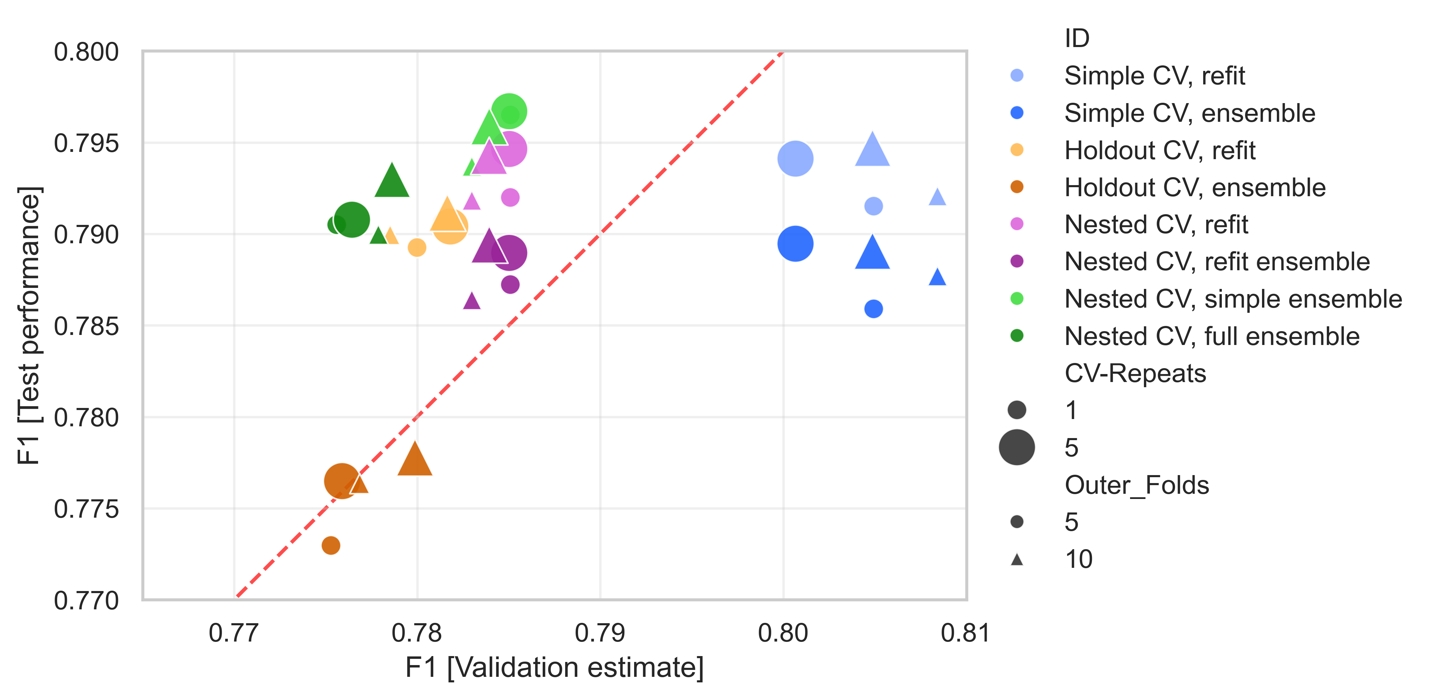

CV schemes below the dashed line indicate overestimation, while those above suggest underestimation of the true performance. Schemes positioned higher in the plot demonstrate better performance on the test set, whereas those positioned lower exhibit poorer performance.

**Figure S8** Scatter plot of the MCC comparing validation and test estimates (amount of overfitting) for the UCI datasets.
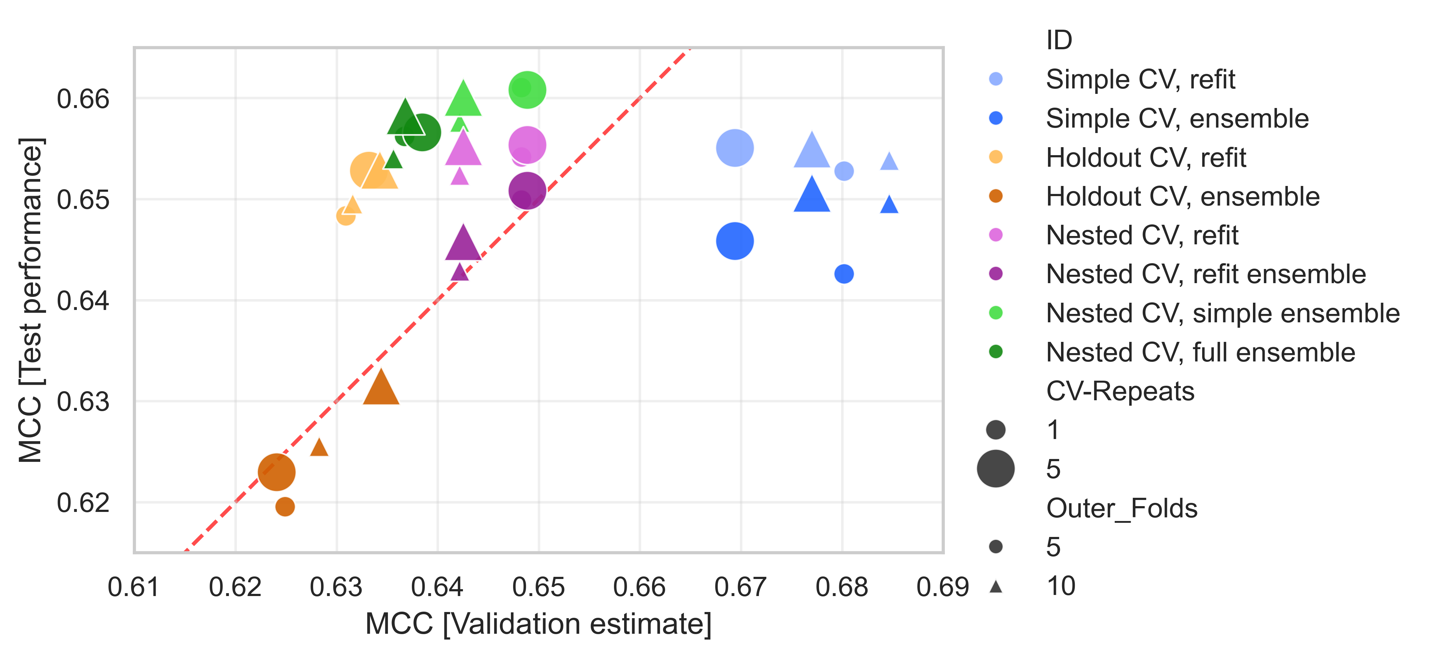

CV schemes below the dashed line indicate overestimation, while those above suggest underestimation of the true performance. Schemes positioned higher in the plot demonstrate better performance on the test set, whereas those positioned lower exhibit poorer performance.

**Figure S9** Association between the amount of overestimation in AUC and dataset characteristics for the UCI datasets.


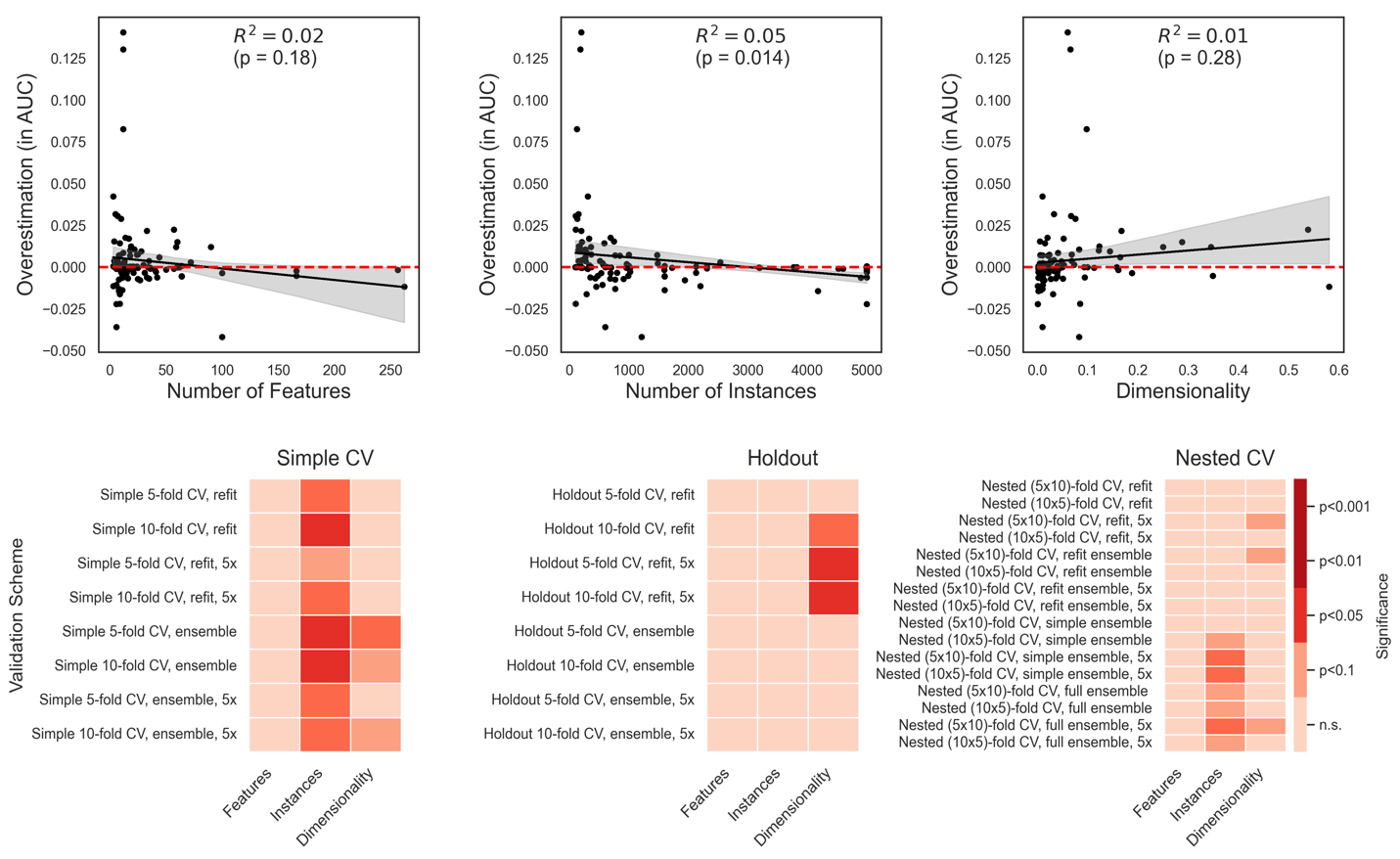


Top row: Scatter plots for simple 5-fold CV demonstrating a significant association between the amount of overestimation in AUC and the number of instances. Bottom row: Heatmaps summarizing the statistical significance (p-values) of the association between overestimation and dataset characteristics across all validation schemes. The three scatter plots shown in the top row correspond to the first row (‘Simple 5-fold CV, refit’) of the ‘Simple CV’ heatmap. For improved clarity, two datasets, Brancato2023 and UPENN-GBM, both of which have a dimensionality greater than 40, were excluded from the plots involving dimensionality.

**Figure S10** Association between the amount of overestimation in F1-score and dataset characteristics for the UCI datasets


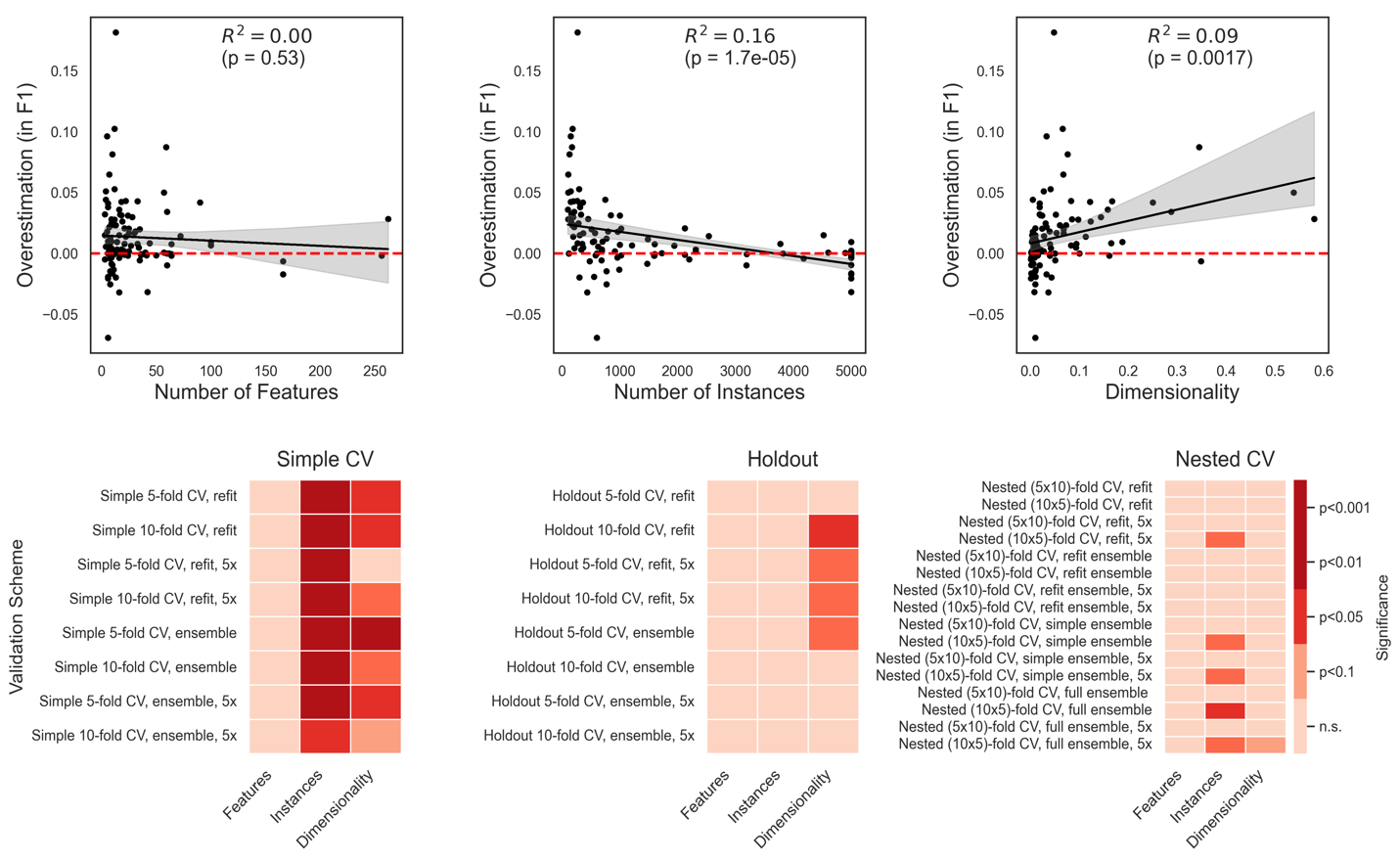


Top row: Scatter plots for simple 5-fold CV demonstrating a significant association between the amount of overestimation in F1-score and the number of instances. Bottom row: Heatmaps summarizing the statistical significance (p-values) of the association between overestimation and dataset characteristics across all validation schemes. The three scatter plots shown in the top row correspond to the first row (‘Simple 5-fold CV, refit’) of the ‘Simple CV’ heatmap. For improved clarity, two datasets, Brancato2023 and UPENN-GBM, both of which have a dimensionality greater than 40, were excluded from the plots involving dimensionality.

**Figure S11** Association between the amount of overestimation in MCC and dataset characteristics for the UCI datasets. For each dataset and validation scheme, the standard deviation across the 25 repeats were computed and plotted.

**
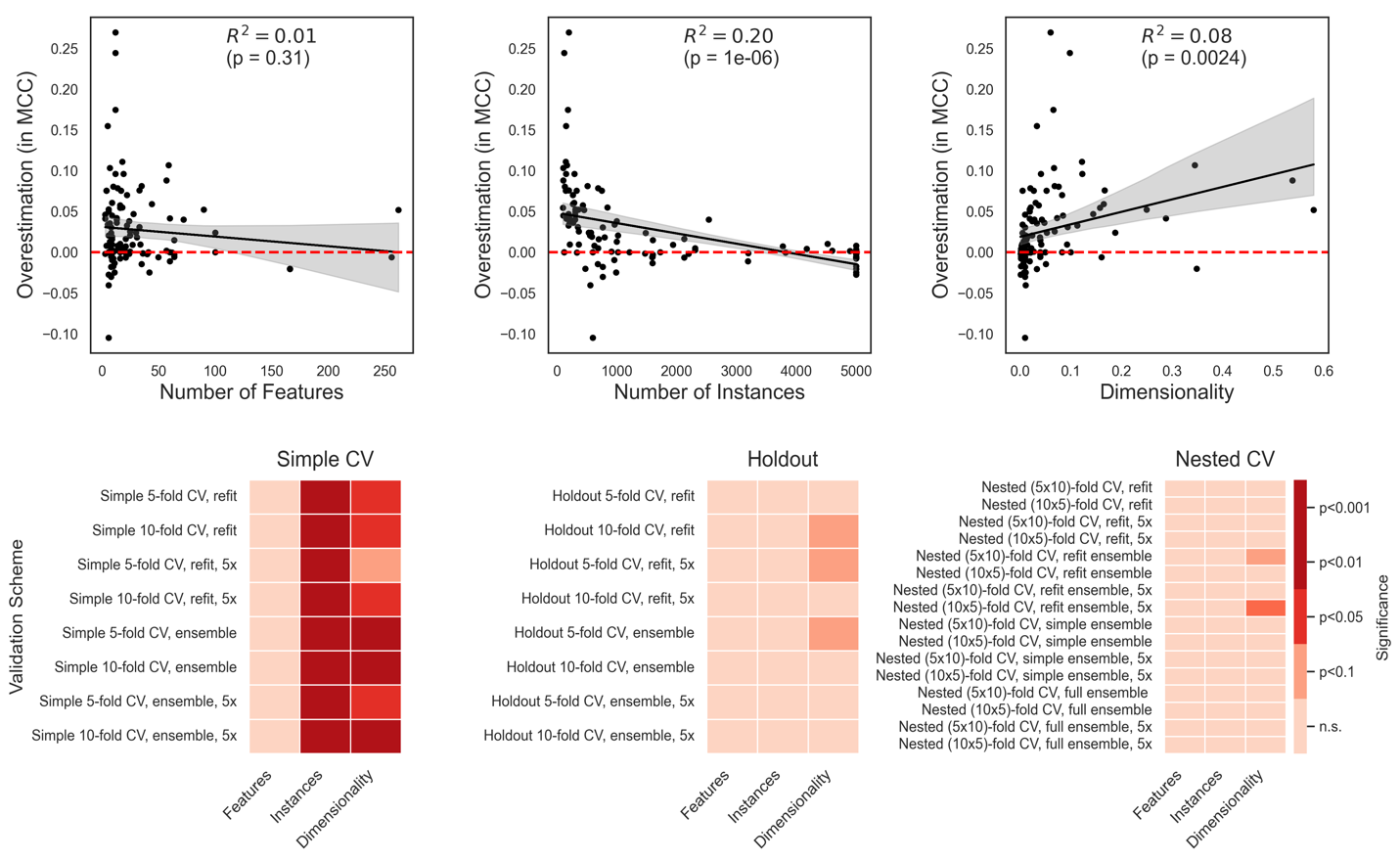
**

Top row: Scatter plots for simple 5-fold CV demonstrating a significant association between the amount of overestimation in MCC and the number of instances. Bottom row: Heatmaps summarizing the statistical significance (p-values) of the association between overestimation and dataset characteristics across all validation schemes. The three scatter plots shown in the top row correspond to the first row (‘Simple 5-fold CV, refit’) of the ‘Simple CV’ heatmap. For improved clarity, two datasets, Brancato2023 and UPENN-GBM, both of which have a dimensionality greater than 40, were excluded from the plots involving dimensionality.

**Figure S12** Graphical boxplot of the standard deviation of the overestimation amount in AUC for the UCI datasets.

**
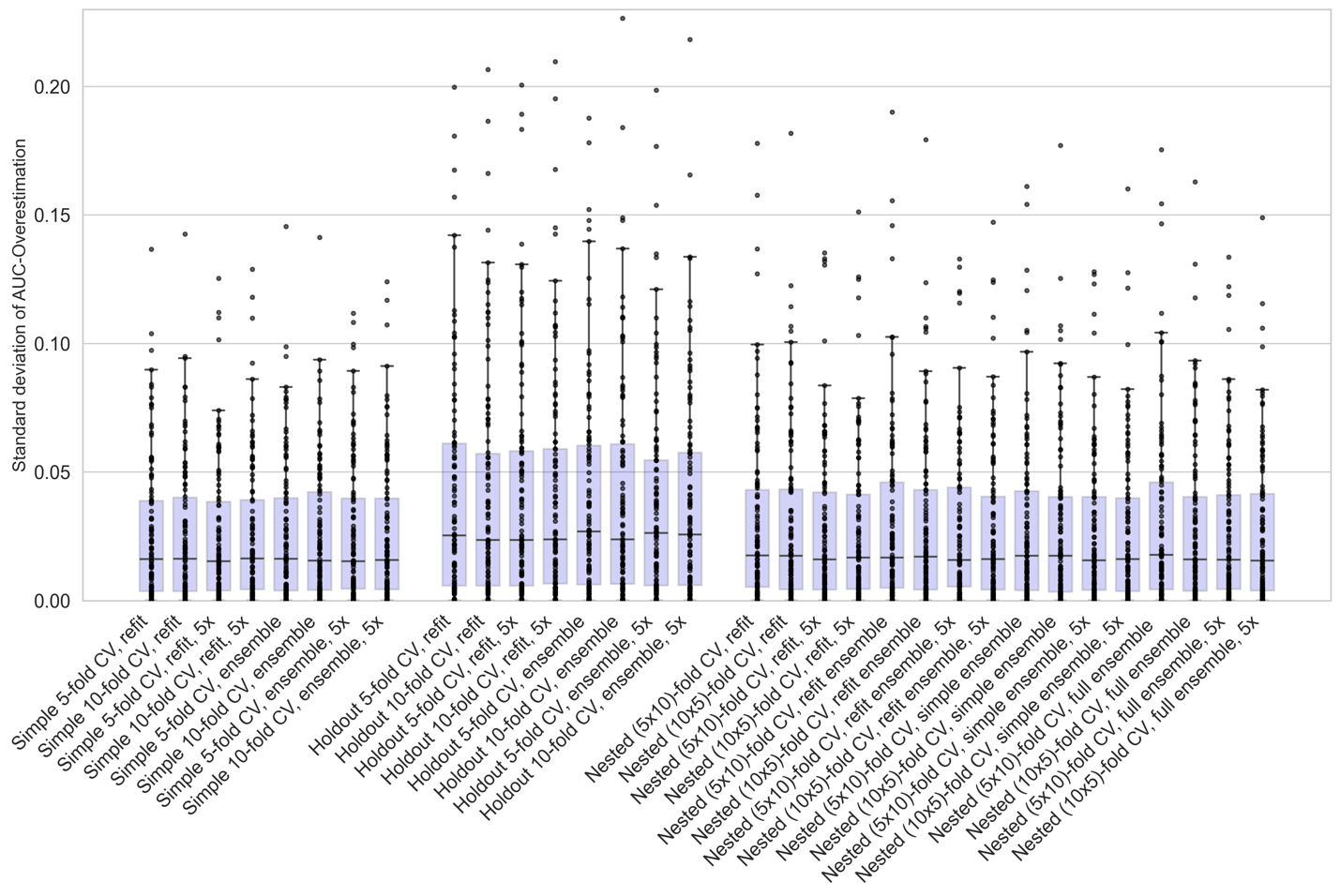
**

For each dataset and validation scheme, the standard deviation across the 10 repeats were averaged and plotted.

**Figure S13** Graphical boxplot of the standard deviation of the overestimation amount in F1 scores for the UCI datasets.

**
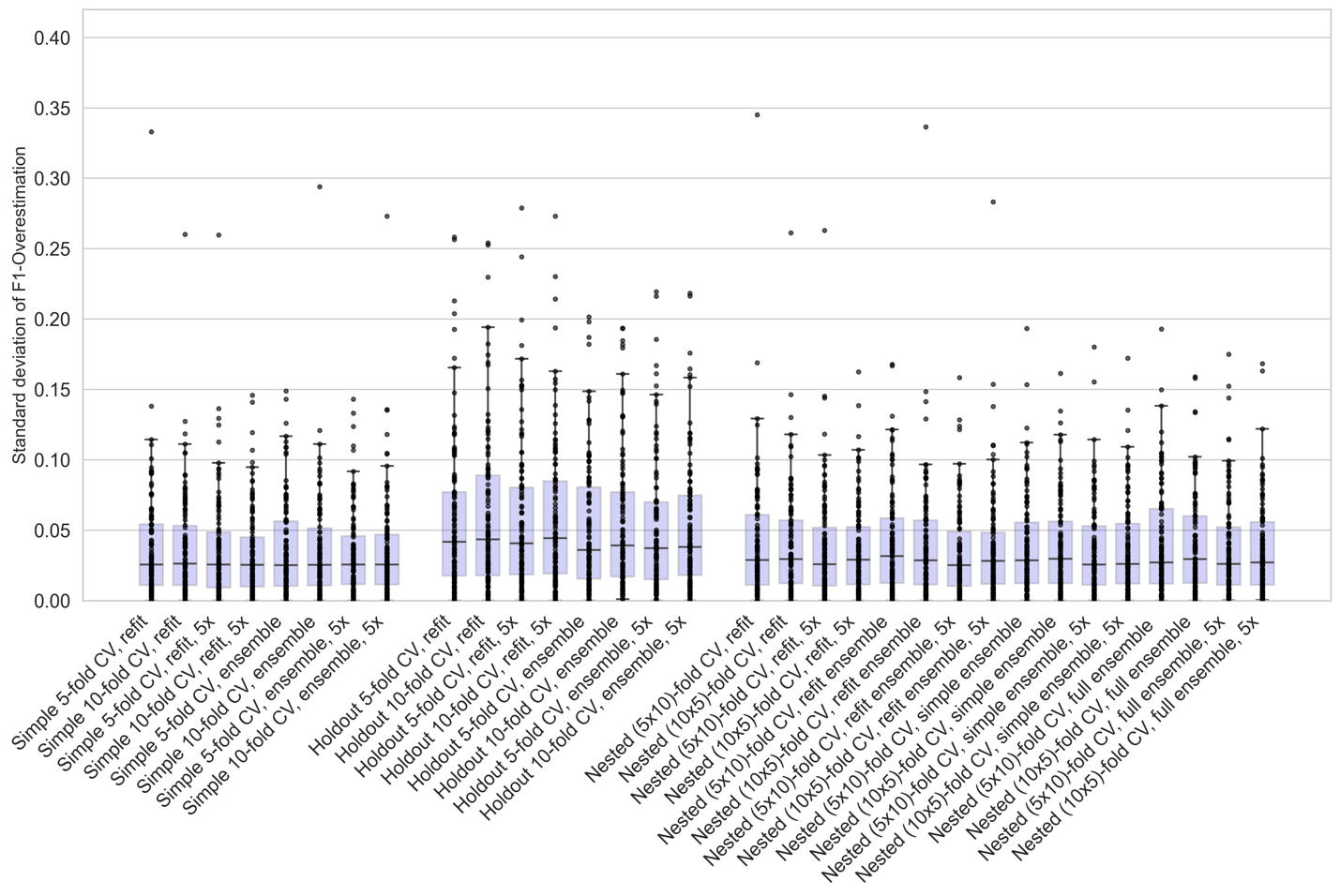
**

For each dataset and validation scheme, the standard deviation across the 10 repeats were averaged and plotted.

**Figure S14** Graphical boxplot of the standard deviation of the overestimation amount in MCC for the UCI datasets
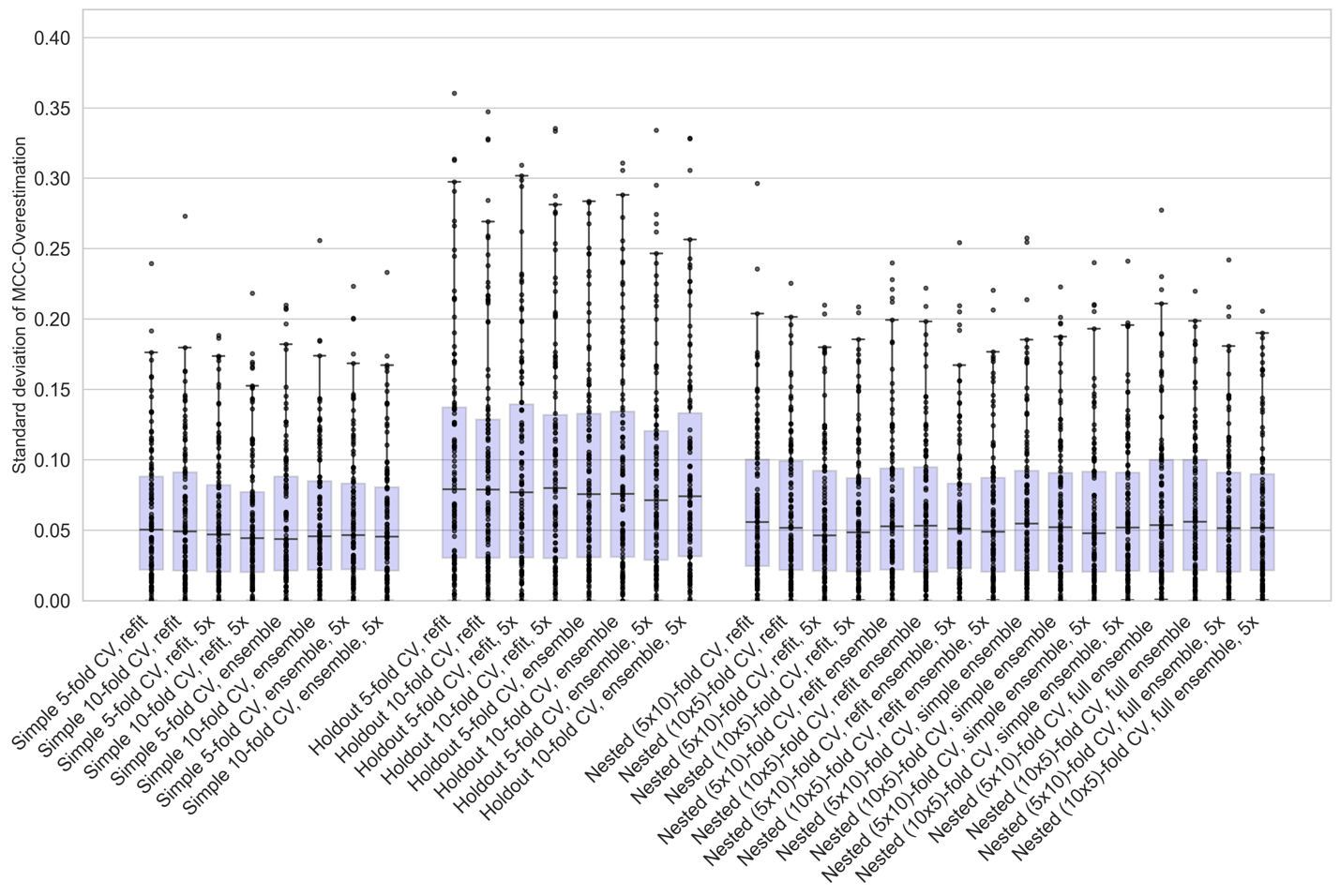


For each dataset and validation scheme, the standard deviation across the 10 repeats were averaged and plotted.

**Figure S15** Association between the standard deviation of the amount of overestimation in AUC and dataset characteristics for the UCI datasets.


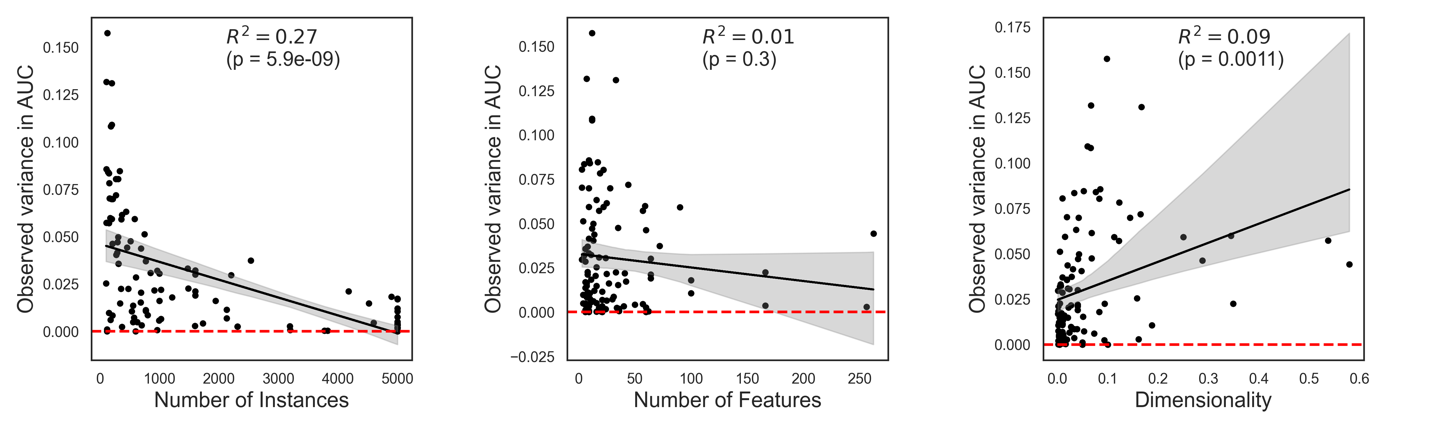


A significant association was observed between the overestimation amount across all metrics and the number of instances, but not with the number of features or the dimensionality

**Figure S16** Association between the standard deviation of the amount of overestimation in F1-score and dataset characteristics for the UCI datasets

**
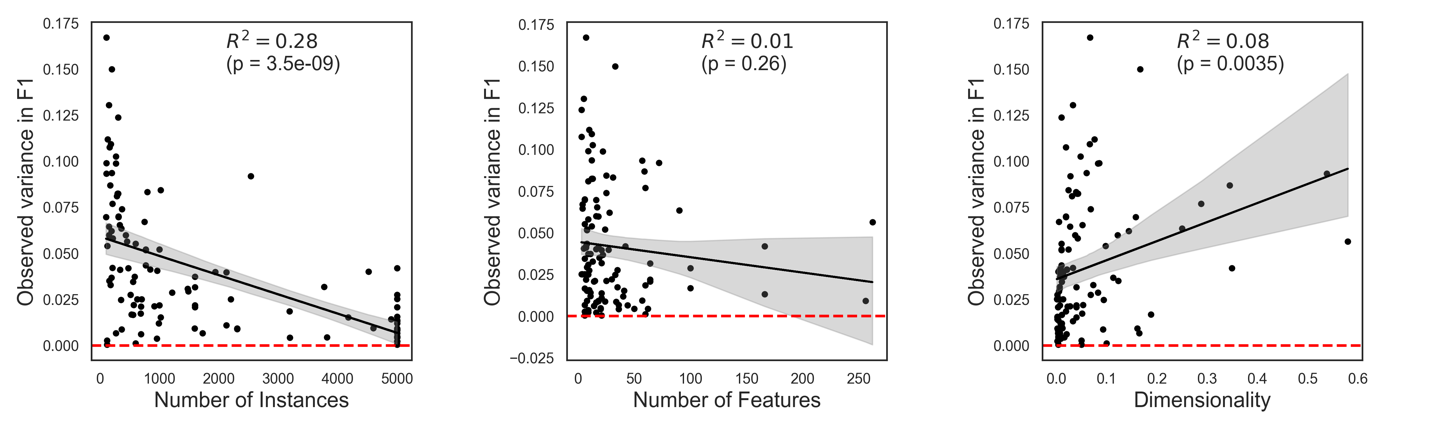
**

A significant association was observed between the amount of overestimation across all metrics and the number of instances, but not with the number of features or the dimensionality

**Figure S17** Association between the standard deviation of the amount of overestimation in MCC and dataset characteristics for the UCI datasets

**
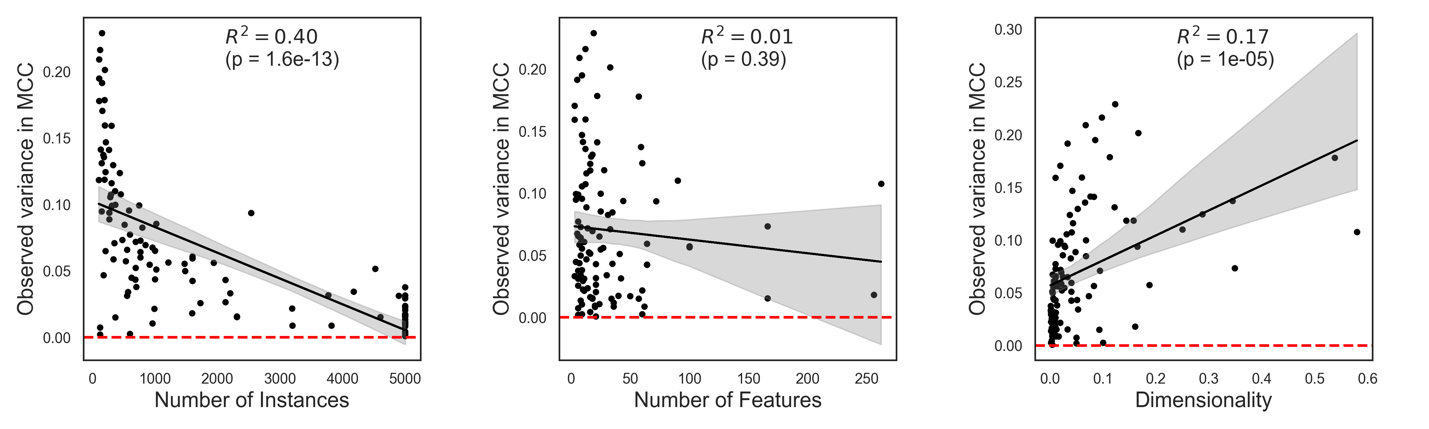
**A significant association was observed between the amount across of overestimation all metrics and the number of instances, but not with the number of features or the dimensionality


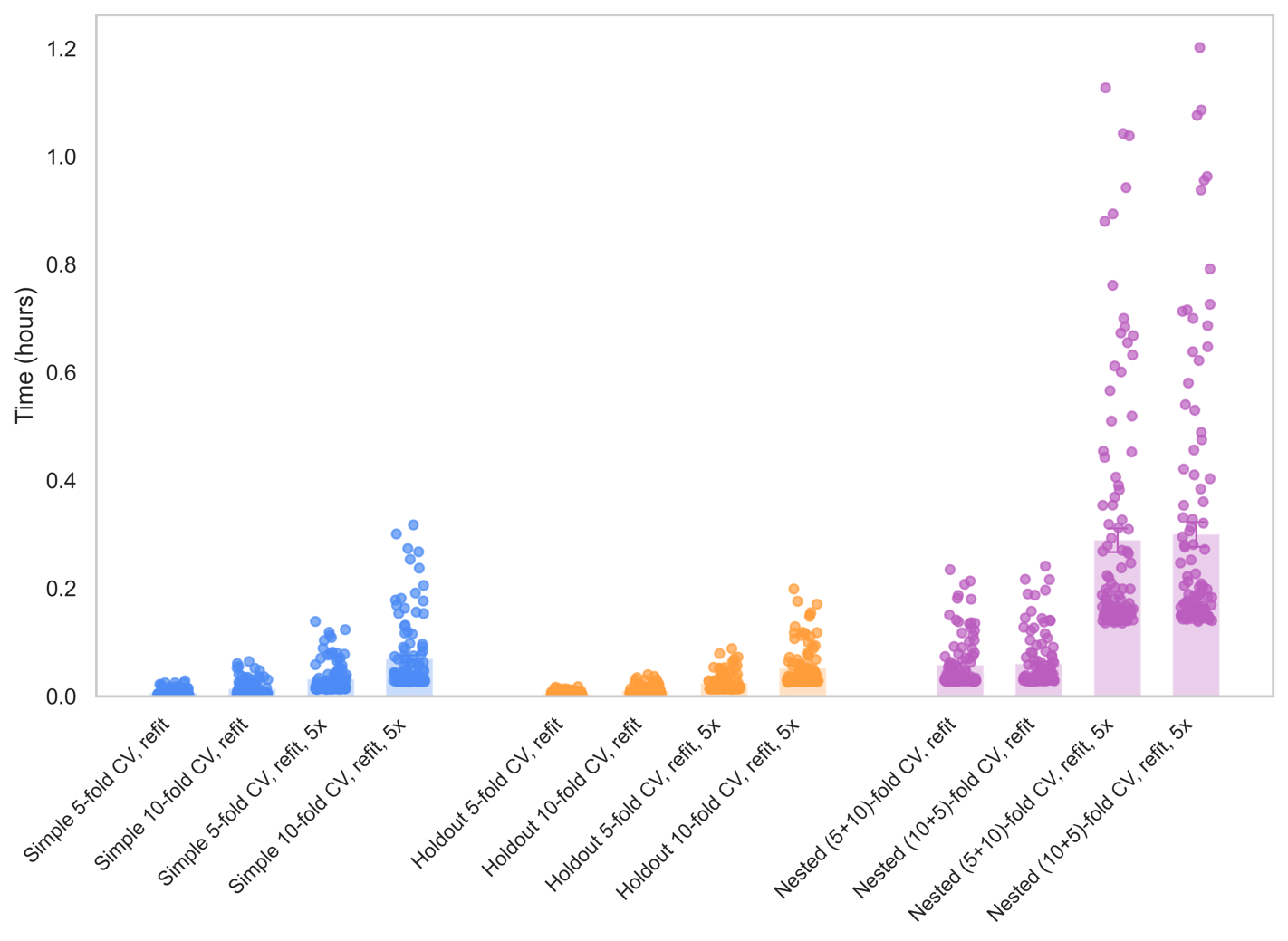
**Figure S18** Graphical plot of the computation times of the validation schemes for the UCI datasets across all metrics.

Scatter and bar plot of the computation times of the validation schemes employed. Each dot denotes the time taken of one repeat over all datasets across all three metrics.

.
